# Sexual intraspecific recombination but not *de novo* origin governs the genesis of new apomictic genotypes in *Potentilla puberula* (Rosaceae)

**Published:** 2018-12-01

**Authors:** Flavia Domizia Nardi, Christoph Dobeš, Dorothee Müller, Tobias Grasegger, Tuuli Myllynen, Henar Alonso-Marcos, Andreas Tribsch

**Affiliations:** 1 Austrian Research Centre for Forests, Department of Forest Genetics, Seckendorff-Gudent-Weg 8, 1131 Vienna, Austria; 2 University of Salzburg, Department of Biosciences, Hellbrunnerstraße 34, 5020 Salzburg, Austria

**Keywords:** AFLP, apomixis, autopolyploidy, cpDNA, origin, *Potentilla*

## Abstract

Apomixis – asexual reproduction via seeds – might arise *de novo* following polyploidisation events, or via reproductive transfer of apomixis. Both processes can be obtained within species or via hybridisation. We aimed to determine the origin of apomictic genotypes in *Potentilla puberula*, a rosaceous species showing reproductive differentiation with ploidy: sexual tetraploids and apomictic penta- to octoploids, which regularly co-occur in sympatry. The study is based on 726 individuals, comprising all cytotypes, collected from 138 populations in the Eastern European Alps. We established relationships of cytotypes based on AFLP fingerprinting and cpDNA sequencing to test (1) whether the apomicts are of recurrent allopolyploid origin or originated from within the species via autopolyploidy, and (2) whether there are indications for reproductive transfer versus *de novo* origin of apomixis. Three principal pathways were identified which explain the origin of new apomictic genotypes, all involving at least one apomictic parent and thus compatible with the idea of reproductive transfer of the apomictic trait to the progeny: (1) self-fertilisation of unreduced egg cells in apomicts; (2) cross-fertilisation among apomicts; and (3) occasionally, heteroploid crosses among sexuals and apomicts. Autopolyploids derived from tetraploid sexuals were repeatedly observed, but did not express apomixis. Finally, our results suggest no role of other species in the origin of extant apomictic genotypes of *P. puberula*, although local hybrids with *P. crantzii* were identified. In conclusion, our results show that the formation of new apomictic genotypes required a genetic contribution from at least one apomictic parent. This finding is in accordance with the idea that apomixis is inheritable in *P. puberula*. On the contrary, lack of apomixis in penta- and hexaploids derived from sexual backgrounds did not support the hypothesis of a *de novo* origin of apomixis. Relatively high frequency of remnant sexuality in the apomicts involving different cytological pathways of seed formation can explain their high cytological and genotypic diversity. Finally, lack of global introgression from a third taxon is in support of *P. puberula* as a concise, although highly diverse, species.

## Introduction

Gametophytic apomixis (henceforth, “apomixis”) refers to reproductive pathways of seed formation in flowering plants which arise through modification of the normal sexual pathway (Rutishauser, 1969; Koltunow & Grossniklaus, 2003). These pathways include the circumvention or interruption of meiosis (i.e., apomeiosis) and the development of egg cells into an embryo without fertilisation (i.e., parthenogenesis; Asker, 1980; Asker & Jerling, 1992). Although rare examples of diploid apomictic taxa are known (*Boechera* Á.Löve & D.Löve: Böcher, 1951; Dobeš & al., 2006; and *Paspalum* L.: Siena & al., 2008), the great majority is polyploid (Asker & Jerling, 1992; Carman, 1997; Comai, 2005; Dickinson & al., 2007). Most apomicts are of allopolyploid origin (Asker & Jerling, 1992) and tend to form amphi-apomictic hybrid complexes with sexual diploids (e.g., Böcher, 1951; Asker, 1970; Campbell & Wright, 1996; Bayer, 1997; Hörandl & Gutermann, 1999). Sexual diploidautopolyploid apomictic complexes, however, do also exist (e.g., in *Paspalum*: Hojsgaard & al., 2008; *Ranunculus* L.: Cosendai & al., 2011; *Townsendia* Hook.: Thompson & Whitton, 2006). In rare cases, sexual-apomictic differentiation further occurs at the polyploid level (Savidan & al., 2001; Rotreklová & al., 2002; Dobeš & al., 2013b).

Several hypotheses have been coined on the functional basis of apomixis. It was proposed to (1) have a genetic basis and originate from co-selection of genes coding for the expression of apomeiosis and parthenogenesis, respectively (Savidan, 1982; Nogler, 1984; Van Dijk & Vijverberg, 2005). Apomeiosis and parthenogenesis may be unlinked and segregate independently (Van Dijk & al., 1999; Noyes & Rieseberg, 2000; Matzk & al., 2005) or could be inherited as monogenic Mendelian trait (Grimanelli & al., 2001; Grossniklaus & al., 2001; Ozias-Akins & Van Dijk, 2007; Hand & Koltunow, 2014). Alternatively, apomixis might result from (2) ectopic expression of the normal sexual pathway, which might be directly linked to genome duplication (i.e., nucleotypic effect) or to the presence of divergent genomes (often allopolyploidy) with asynchronous reproductive gene expression (the “duplicate-gene asynchrony hypothesis”, Carman, 1997; Sharbel & al., 2010).

Apomixis may originate *de novo* independently multiple times in taxa or lineages preadapted for the evolution of this reproductive mode. This is shown clearly by the great diversity of apomictic pathways (Asker & Jerling, 1992) and their polyphyletic distribution among the angiosperm orders (Carman, 1997; Hörandl & Hojsgaard, 2012). Rates of *de novo* origins, however, can be assumed to be constrained by the developmental and genetic complexity of the trait. Additionally, the establishment of a functional apomict may require several generations (Van Dijk & Vijverberg, 2005; Hojsgaard & al., 2014). Alternatively, apomixis may be transferred to sexuals via introgression by their apomictic relatives (Van Dijk & Vijverberg, 2005). While egg cells from apomictic individuals are usually unreduced, their pollen is often meiotically reduced and at least partially viable (Van Dijk, 2003; Mogie & al., 2007). Thus, pollen from apomicts is able to fertilise egg cells produced by sexual individuals (Mogie & al., 2007). Since apomixis has been shown to be inheritable (Asker, 1980; Savidan, 1982; Nogler, 1984; Grossniklaus & al., 2001; Ozias-Akins & Van Dijk, 2007), such cross-fertilisations may lead to the formation of new apomictic clonal lineages. Hybridisation between apomicts and sexual relatives was repeatedly found, for instance in *Boehmeria spicata* Thunb. (Yahara, 1990) and in *Taraxacum* sect. *Ruderalia* Kirschner & al. (Menken & al., 1995). Extensive introgression and hybridisation might also be responsible for the interspecific transfer of apomixis in *Bothriochloa* Kuntze and closely related genera (De Wet & Harlan, 1970). To give another example, Lo & al. (2010) revealed that apomictic tetraploids of *Crataegus suksdorfii* (Sarg.) Kruschke originated from backcrossing of allotriploids to sexual diploids.

Geographical separation, which often occurs in amphiapomictic complexes in the form of geographical parthenogenesis (Bierzychudek, 1985; Hörandl, 2006), represents a barrier of gene flow among sexuals and apomicts. In accordance with this argument, it has been shown that apomictic populations which at least partly occur with sexuals in sympatry are often genotypically more diverse than fully allopatric ones (Hörandl & al., 2001; Daurelio & al., 2004; Hörandl & Paun, 2007). This observation suggests repeated recent origins of apomicts. Geographical parthenogenesis might be also the result of following reproductive interactions among sexuals and apomicts (Cosendai & Hörandl, 2010; Hülber & al., 2013; Kirchheimer & al., 2018). Unidirectional fertilisation of sexuals by apomicts and introgression of the apomictic trait into sexual populations may in fact lead to their replacement (Mogie, 1992; Joshi & Moody, 1995, 1998). Thus, study systems in which sexuals and their apomictic descendants occur in sympatry are expected to hold the clue for the understanding of mechanisms governing the evolution and dynamics of amphi-apomictic complexes.

Here we investigate the origin of apomictic genotypes of *Potentilla puberula*, a rosaceous species showing a remarkable differentiation of sexual tetraploids on the one hand and apomictic penta- to octoploids on the other hand. Both reproductive modes occur sympatrically in the Eastern European Alps. *Potentilla puberula* itself has been proposed as an allopolyploid species (Wolf, 1908; Ehrendorfer, 1970; Soják, 2010), and it is already known to be a parental species of *P. alpicola* La Soie, via multiple hybridisation events with hexaploid *P. argentea* L. (Paule & al., 2012). So far, it has remained unclear whether the apomictic cytotypes of *P. puberula* themselves did originate from hybridisation events with other closely related species (allopolyploid origin) or whether they evolved intraspecifically via autopolyploidisation. The latter has been tentatively suggested because of little molecular differentiation among sexual and apomictic cytotypes (Paule & al., 2012). We based this study on established individuals from natural populations and used molecular methods (Amplified Fragment Length Polymorphisms [AFLPs] and plastid DNA [cpDNA] sequencing) for estimating genetic relatedness and structure. In particular, we address the following questions: (1) did apomictic genotypes of *P. puberula* originate via allopolyploidy from (multiple) hybridisation events of sexual genotypes with sympatric related apomictic and/or sexual *Potentilla* L. species? (2) or are the apomicts of intraspecific origin? Particularly we (2a) address the role of sexual recombination involving apomicts in the formation of new apomictic genotypes, and (2b) alternatively, ask whether there is evidence for a (re)current autopolyploid *de novo* origin of apomicts from sexuals. Since we are not able to reconstruct either the individual generations leading to apomixis from established populations/individuals, or the duration of the process, we determine the origin of genotypes in terms of molecular genetic relatedness. If apomicts arose *de novo* from sexual parents, we would expect finding apomictic genotypes being genetically similar to sexuals. On the contrary, we would expect apomictic genotypes deriving by crosses involving other apomicts to be genetically admixed (in case of crosses involving sexuals) or close to other apomictic genotypes (in case of crosses among apomicts).

## Materials and Methods

### Study system

*Potentilla puberula* (Rosaceae; *Potentilla pusilla* Host; Soják, 2010) is a herbaceous species inhabiting mainly foothills to montane (rarely subalpine) xeric grasslands on shallow soil ranging from the Eastern European Alps to the Western Carpathians (Kurtto & al., 2004). Five ploidy cytotypes are known: tetraploids (*x* = 7, 2*n* = 28), pentaploids (2*n* = 35), hexaploids (2*n* = 42), heptaploids (2*n* = 49), and octoploids (2*n* = 56), although sporadic higher ploidy levels have been found as well (Dobeš, 1999). Tetraploids reproduce almost always sexually and are self-incompatible, whereas the other cytotypes are usually pseudogamous apomictic (i.e., the endosperm needs a preceding fertilisation event initiating seed development) and are self-compatible (Dobeš & al., 2013b). No embryological observations are explicitly available for apomictic *P. puberula* (but see Håkansson, 1946 for the embryology of a sexual tetraploid cytotype). However, both diplospory and apospory have been documented for *P. tabernaemontani* Asch. (e.g., Smith, 1963a), a taxon of a formerly used broader circumscription including our species (Meusel & al., 1965; Kurtto & al., 2004).

Ex-situ crossing experiments revealed homoploid pollen preference (Alonso-Marcos & al., 2018) and reduction of the seed set resulting from heteroploid crosses in the tetraploids (Dobeš & al., 2018). However, viability of pollen from apomicts, together with the insignificancy of the mentor effect and the evidence that seeds from heteroploid crosses are still produced (Dobeš & al., 2018) – even in presence of competitive homoploid pollen (Alonso-Marcos & al., 2018) – suggest that natural fertilisation of tetraploids by apomicts is possible.

### Plant material

A total of 2038 individuals of *P. puberula* were collected from 138 populations in the Eastern Alps (Appendix 1).

Six additional species closely related to *P. puberula* (Wolf, 1908; Dobeš & Paule, 2010; Paule & al., 2012) and occurring in sympatry in the study area with the species (Kurtto & al., 2004) were included to test for their possible role in the origin of apomictic genotypes: *Potentilla argentea* L., *P. aurea* L., *P. brauneana* Hoppe, *P. crantzii* (Crantz) Beck ex Fritsch, *P. frigida* Vill., *P. grandiflora* L. and morphologically intermediate forms of *P. crantzii* × *P. puberula* (Table 1; Appendix 1). In total, 122 individuals from 36 populations were sampled. Three species are known to reproduce sexually (Dobeš & al., 2015) at the di- (*P. aurea*) and tetraploid level (*P. frigida*, *P. grandiflora*). No evidence on reproductive mode is available for *P. brauneana* yet, but diploidy suggests that it reproduces sexually since apomixis is bound to polyploidy in the genus *Potentilla* (Dobeš & al., 2015). *Potentilla argentea* and *P. crantzii* show reproductive differentiation among their cytotypes. Diploid *P. argentea* reproduces sexually, whereas its hexaploid cytotype is preferentially apomictic (Paule & al., 2011; Dobeš & al., 2015). In *P. crantzii*, tetraploids are sexual (Czapik, 1961, 1962) and higher ploidy cytotypes preferentially apomictic (Smith, 1963a, b). *Potentilla argentea* is repeatedly found growing together with *P. puberula* in the study area, *P. aurea* and *P. crantzii* get occasionally in contact with *P. puberula* at higher elevations, while the remaining species hardly get into direct contact with *P. puberula* due to their preference for alpine to nival habitats (Wolf, 1908; Polatschek, 2000; Kurtto & al., 2004). Additionally, 15 individuals of *Potentilla incana* G.Gaertn. & al., which is a sexual tetraploid in Central Europe (Dobeš & al., 2015), from 3 populations in eastern Austria and 3 individuals from a single population at the southern limit of our study area were included in the analysis as a reference (Appendix 1). This species is closely related to *P. puberula*, both belonging to the *P. verna* L. agg. (Wolf, 1908; Ehrendorfer, 1973). Herbarium vouchers were submitted to the herbaria of the Natural History Museum of Vienna (W) and of the University of Göttingen (GOET; Appendix 2 and Electr. Suppl. 1: Table S1).

### DNA ploidy level estimation

DNA ploidy was determined for all 2038 individuals of *P. puberula*, as well as 52 individuals of other species with multiple ploidy levels (i.e., *P. argentea* and *P. crantzii*). We used a Partec ML Ploidy Analyser (Partec, Münster, Germany) and followed the protocol of Doležel & al. (2007): leaf material was chopped together with the internal standard *Solanum pseudocapsicum* L. (2C-value = 2.59 pg, Temsch & al., 2010) in 500 μl Otto I buffer (Otto, 1990), filtered through a 20 μm nylon mesh (Partec CellTrics, Partec, Münster, Germany) and stained with 1 ml Otto II buffer (Otto, 1990) containing 4 μg DAPI (4′-6-diamidino-2-phenylindole). The mean fluorescence intensities of sample and standard from each measurement were calculated using FloMax v.2.9 (Quantum Analysis, 2014). The ploidy of each individual was estimated calculating the sample/standard fluorescence ratio and using plants of known ploidy level as karyological reference (Ptl4048, 2*n* = 4*x* = 28; Ptl4184, 2*n* = 5*x* = 35; Ptl4133, 2*n* = 7*x* = 49; Paule & al., 2012).

### Reproductive mode screening

The reproductive origin of 501 fruitlets (referred to as seeds in the following) from 464 individuals of *P. puberula* was determined via flow cytometric seed screen (FCSS, Matzk & al., 2000). Since apomictic-sexual reproductive differentiation in *P. puberula* is strong both at the level of cytotypes and individuals (Dobeš & al., 2013b, 2018), one seed per individual was generally measured. However, exceptionally, sexual penta- to octoploids were also observed. When a seed from a penta- to octoploid individual was estimated to be sexually derived, we, therefore, analysed more seeds at the individual level. Seeds were chopped in 300 μl Otto I buffer (Otto, 1990) together with *Pisum sativum* L. ‘Kleine Rheinländerin’ (Greilhuber & Ebert, 1994) and placed 30 min on ice. The material was then ultrasonicated for 1 min, filtered through a 20 μm mesh filter (Partec CellTrics) and finally stained in 1200 μl Otto II buffer (Otto, 1990) containing 0.2 μg/ml DAPI. After 15 min, the samples were measured with a Partec ML Ploidy Analyser. The mean fluorescence intensities of embryo, endosperm and standard from each measurement were calculated using FloMax v.2.9 (Quantum Analysis, 2014).

The FCSS method is based on different ploidy levels of endosperm and embryo occurring in a seed: in most angiosperms, a triplophasic endosperm is formed by fertilisation of two haplophasic polar nuclei. The ploidy ratio among endosperm and embryo (i.e., the peak index) thus differs depending on the reproductive mode of seed formation: in regular sexuality a diplophasic embryo (1*n* + 1*n*) and a triplophasic endosperm (2*n* + 1*n*) are expected, whereas in pseudogamous apomixis a diplophasic embryo (2*n* + 0*n*) and a penta- or hexaphasic endosperm (4*n* + 1–2*n*, depending on the male gamete) are usually formed, resulting in peak indices of respectively 3/2 and 5(or 6)/2 (Matzk & al., 2000). However, these ratios only apply under the assumption of equal ploidy of parents, regularity of meiosis, and functional male meiosis, which do not necessarily apply outside a controlled crossing study. We therefore allowed for continuous variation of peak indices and inferred the origin of the megagametophyte calculating the female genomic contribution: peak indices lower and higher than 2 indicate a zygotic and a parthenogenetic origin, respectively (Dobeš & al., 2013a). However, an endosperm with a ploidy twice that of the embryo is practically often indistinguishable from the embryo’s G2 phase; therefore, we considered only peak indices < 1.9 and > 2.1. The maternal genomic contribution, calculated according to Dobeš & al. (2013a), was used to infer the megagametophyte origin: megagametophyte ploidies equal to the mother plant ploidy and equal to its half indicated an apomeiotic and a meiotic megagametophyte development, respectively. The combination of the two categories led to the identification of four main reproductive pathways: regular sexuality (meiosis and fertilisation), irregular sexuality (here defined as apomeiosis followed by fertilisation and giving rise to B_III_ hybrids, Rutishauser, 1948; or S_III_ progeny in case of selfing, Bicknell & al., 2003), haploid parthenogenesis (meiosis and parthenogenesis) and apomixis (apomeiosis and parthenogenesis; Table 2). The reproductive origin of additional 201 seeds from 38 individuals from populations 9, 13, 15 and 17 was taken from Dobeš & al. (2013b).

### DNA extraction, cpDNA amplification and sequencing

For all molecular analyses, four individuals per cytotype per population (726 of *P. puberula* and a total of 140 of the other taxa) were randomly selected. Genomic DNA was extracted from silica gel-dried leaves taken directly in the field using the CTAB method following the protocol by Doyle & Doyle (1987) with minor modifications: ground leaf material was washed twice in Sorbitol buffer (100 mM Tris-Cl pH 8.0, 0.35 M Sorbitol, 5 mM EDTA, 1% PVP-40) and incubated for 10 min at 65°C in 3× CTAB buffer (100 mM Tris-Cl pH 8.0, 3 M NaCl, 20 mM EDTA, 3% CTAB, 2% PVP-40) and 2 μl RNase A (10 mg/ml, Thermo Scientific, Massachusetts, U.S.A); at the end of the extraction, the DNA pellet was washed in 70% ethanol and resuspended in ddH_2_O.

For each individual of *P. puberula*, the plastid *trnH(gug)*-*psbA* intergenic spacer (IGS) and *rps16* intron were amplified using the primers trnH(gug) and psbA (Aldrich & al., 1988; Shaw & al., 2005) and rpS16F and rpS16R (Oxelman & al., 1997; Shaw & al., 2005) respectively. PCR reactions were performed in 30 μl master mix containing 5× Green GoTaq buffer (Promega, Madison, Wisconsin, U.S.A.), 0.2 μM of each primer, 0.2 mM dNTP mix, 0.83 U GoTaq DNA polymerase (Promega), and approximately 10 ng of template DNA using a My Cycler (Bio-Rad Laboratories, Vienna, Austria) thermal cycler. Thermal cycling started with a denaturation step at 95°C lasting 2 min, followed by 35 cycles each of 30 s denaturation at 95°C, 30 s annealing at 50°C, 1 min 20 s elongation at 72°C, and lasted with 5 min final elongation at 72°C and a final hold at 4°C.

The sequences of the two cpDNA markers were edited and combined on Geneious v.8.0 (Kearse & al., 2012; Biomatters, 2014). Haplotypes from the uncut sequences were collapsed using FaBox v.1.41 (Villesen, 2007) and deposited in GenBank (Electr. Suppl. 1: Table S2; Benson & al., 2005).

### AFLP fingerprinting

Individuals selected for the molecular analyses (726 of *P. puberula* and a total of 140 of the other taxa) – including replicated samples following Bonin & al. (2004) – were genotyped using AFLP fingerprinting according to the protocol established by Vos & al. (1995) with the following modifications: approximately 300 ng of DNA was digested and ligated in a 11 μl reaction mix containing 10× T4-ligase buffer, 68.7 mM NaCl, 30.8 μg BSA, 0.9 U T4-ligase (Promega), 1 U MseI (New England Biolabs, Ipswich, Massachusetts, U.S.A.), 8 U EcoRI (Promega), and 6.25 μM MseI-adaptor and 0.63 μM EcoRI-adaptor. The reaction mix was incubated for 3 h at 37°C, and the restriction-ligation product was subsequently diluted 1 : 20.

In the pre-selective PCR, 2 μl of the diluted restrictionligation product was used in a total reaction volume of 10 μl, containing: 5× Green GoTaq Buffer (Promega), 0.29 mM dNTP mix, 0.38 μM EcoRI-A primer (5′-GAC TGC GTA CCA ATT CA-A-3′), 0.38 μM MseI-C primer (5′-GAT GAG TCC TGA GTA AC-C-3′), and 0.13 U GoTaq G2 polymerase (Promega). The reactions were held at 72°C for 2 min followed by 30 cycles of: 94°C for 30 s, 56°C for 30 s, and 72°C for 1 min, with a final extension at 72°C for 10 min.

For the selective PCR, 2 μl of 1 : 20-diluted pre-selective PCR product was used as a template in three different reactions including differently labelled primer combinations, for a reaction volume of 10 μl each containing: 5× Green GoTaq Buffer (Promega), 0.28 mM dNTP mix, 0.34 μM EcoRI-fluorescence-labelled primer, 0.34 μM MseI primer (EcoRI-AGG [VIC]/MseI-CTC, EcoRI-AAC [6-FAM]/MseI-CTT, EcoRI-AGC [NED]/MseI- CTG), and 0.2 U GoTaq G2 polymerase (Promega). The reactions were held at 94°C for 2 min followed by 10 cycles of: 94°C for 20 s, 66°C → 57°C (–1°C per cycle) for 30 s and 72°C for 2 min, followed by 20 cycles of: 94°C for 20 s, 56°C for 30 s and 72°C for 2 min, with a final 30 min extension at 60°C. The product of each step (restrictionligation, pre-selective and selective amplifications) was run on 1% agarose gel together with a negative control.

For each sample, the three different PCR products were combined, and the fragments were separated on a MegaBACE 1000 DNA capillary-sequencer together with an ET-ROX 400 size standard (GE Healthcare Biosciences, Pittsburgh, Pennsylvania, U.S.A.). In each run, 48 samples including negative and carryover controls (Bonin & al., 2004) were analysed. Raw data were visualised and quality checked with Fragment Profiler v.1.2 (Amersham Biosciences, Amersham, U.K.) and the fragments manually scored as binary presence-absence data using DAx v.9.0 (Van Mierlo Software Consultancy, Eindhoven, The Netherlands) and following the procedure described by Bendiksby & al. (2011).

Two final presence-absence matrices were created by two different scoring procedures: to have a higher resolution at the specific level, all the 726 individuals of *P. puberula* only were included (intraspecific dataset), and to better identify species-specific markers, a subset of 163 individuals of *P. puberula* covering the whole sampling area and all ploidy levels (Appendix 1) was scored together with all 140 samples of the other taxa (interspecific dataset).

### Data analyses

Since genotyping errors might overestimate the genotypic differences among genotypes (Bonin & al., 2004), a threshold was estimated from both the error rate (Pompanon & al., 2005) and the distributions of pairwise genotypic distances within populations of individuals of the same ploidy, calculated with the R script AFLPdat v.2010 (Ehrich, 2006; R Development Core Team, 2018). AFLP phenotypes showing pairwise distances not exceeding the threshold were merged into unique genotypes (“genotype” in the following) and only those were used in the analyses.

Principal coordinate analyses (PCoAs) calculated with Dice dissimilarity index were run using the R packages ape (Paradis & al., 2004) and vegan v.2.5-2 (Oksanen & al., 2018) to evaluate the genetic similarity among all individuals of *P. puberula* alone (intraspecific dataset) and among all studied species (interspecific dataset).

The Bayesian clustering-based software STRUCTURE v.2.3.4 (Pritchard & al., 2000) was used to detect genetic admixture among groups. To test for introgression from sympatrically occurring species in apomictic individuals of *P. puberula*, we used the admixture model with independent allele frequencies, and 10 replicates were run for each pre-defined number of clusters (K) ranging from 1–10. A burn-in period of 2 × 10^5^ and Markov chain Monte Carlo (MCMC) of 5 × 10^5^ iterations was chosen. Null alleles were defined to account for genotypic ambiguity (Falush & al., 2007). To assess the level of admixture among sexual and apomictic *P. puberula*, we ran a STRUCTURE analysis with ploidy as prior information (USEPOPINFO = 1) for all tetraploids. This function is available only for diploids, thus we renounced to set the individuals as polyploids. Although studies on intraspecific ploidy variation often attempt a differential ploidy definition (Stöck & al., 2010), no simulation has ever been run to test the best way to deal with different ploidy levels (Meirmans & al., 2018) and the superiority of this approach over the standard diploid definition has not been proven yet. On the contrary, the prior information approach – with the limitation of a diploid definition of the data – allows to assist genetic clustering in genetically low-structured groups. This approach is meaningful in our context, because the tetraploid group, reproducing sexually, can be seen as a single genetic population. We thus ran a STRUCTURE analysis with 10 replicates for each K from 1 to 5 with burn-in of 2 × 10^5^ and MCMC of 5 × 10^5^ iterations. Both the admixture and the correlated allele frequencies model were used, and we allowed a migration prior of 0.1. Since a phylogeographical signal was detected in the results, we ran a similar additional analysis including all and only the 105 genotypes from Eastern Tyrol (see Appendix 1), with the following differences: we ran 2 × 10^5^ iterations after a 10^5^ burn-in, and the migration prior was set to 0.05. All markers monomorphic in these selected genotypes were removed. The most likely values of K were estimated from the likelihood distribution of single runs per K value. For each chosen K, the runs with the highest likelihood were chosen and graphically displayed with the R package pophelper v.2.2.7 (Francis, 2017).

All graphs were created in R (R Development Core Team, 2018), using the R package ggplot2 v.3.0.0 (Wickham, 2016).

## Results

### DNA ploidy of adults and reproductive mode screening

Five distinct classes of sample : standard fluorescence ratios were identified for *P. puberula*. The classes corresponded to the tetra- (966 individuals; 47.40 %), penta- (613; 30.08%), hexa- (116; 5.69%), hepta- (280; 13.74%) and octoploid (63; 3.09%) DNA ploidy levels. Of the 138 populations, 77 were of mixed ploidy, the others were uniformly tetra- (40 populations), penta- (17), hexa- (2), or heptaploid (2; Appendix 1). Fourteen individuals (58.33%) of *P. argentea* were determined as diploid, whereas ten were hexaploid (41.67%). One population out of four was cytologically mixed (Appendix 1). Individuals of *P. crantzii* were determined as tetra- (5 individuals; 21.74%), penta- (2; 8.70%), hexa- (8; 34.78%) and heptaploid (8; 34.78%). Two populations out of six were cytologically mixed (Appendix 1). The individual measurements are provided in Table S3 (Electr. Suppl. 2).

The intra-specific reproductive differentiation by ploidy in *P. puberula* observed by Dobeš & al. (2013b) was basically confirmed, with tetraploids forming 97.46% (192) of the seeds by regular sexuality and penta- to octoploids 84.01% (247)of the seeds apomictically (Table 3). Irregular sexuality (B_III_ hybrids) was found in equal percentages in tetraploids (4 seeds, 2.03% of seeds) and penta- to octoploids (6 seeds, 2.04%), whereas haploid parthenogenesis was present exclusively in penta- to octoploids (10 seeds, 3.40% of seeds). Among the higher ploidy levels, 13 hexaploid individuals which were assigned to the tetraploid cluster by the STRUCTURE analysis (see below) produced all seeds (15) by regular sexuality, and no parthenogenetically derived seed was found in the same genetic group (Table 3). On the contrary, 92.02% (369) of seeds produced by individuals assigned to the high ploidy level genetic cluster was apomictically derived, whereas 2.74% (11), 3.74% (15) and 1.50% (6) were derived respectively by haploid parthenogenesis, regular and irregular sexuality (Table 3). For individuals admixed among the two clusters no clear tendency was observed, with 41.67% (5) of their seeds produced by apomixis, 33.33% (4) by regular sexuality and 25.00% (3) by irregular sexuality (Table 3). Sample histograms for each reproductive mode of seed formation are provided in Fig. S1 (Electr. Suppl. 1). Individual measurements are provided in Table S4 (Electr. Suppl. 2).

### Genotypic and haplotypic variation within *P. puberula*

The AFLP scoring of 726 successfully genotyped individuals of *P. puberula* resulted in the identification of 370 polymorphic markers ranging between 62 and 401 bp after removal of non-reproducible and non-informative fragments, with an error rate of 2.35% (the presence-absence matrix is provided as Table S5 in Electr. Suppl. S2). We identified 554 genotypes applying a pragmatic threshold of 12 mismatches, derived from the observed error rate of 2.35% and the distribution of pairwise genotypic distances (Electr. Suppl. 1.: Fig. S2). All tetraploid individuals were represented by unique genotypes (D_GT_ = 1), while 300 unique genotypes representing 471 individuals were found for the penta- to octoploids. The proportion of polymorphic fragments and the number of private fragments was comparable among tetraploids (14 fragments) and the group of high ploidy levels (12 fragments), but the private fragments were largely shared among the high-ploidy cytotypes, since only one private fragment each in the penta- and heptaploids, respectively, remained when cytotypes were considered separately.

Interestingly, 2.71% of the genotypes was shared among cytoypes (Table 4) and involving almost exclusively high-ploidy cytotypes. In particular penta- and hepta-/octoploids, and hexa- and octoploids shared the same genotype.

In total, 719 individuals of *P. puberula* were successfully amplified for both *trnH-psbA* and *rps16* markers (Electr.Suppl. S2: Table S5). The length of the combined sequences ranged from 1108 bp to 1177 bp. Overall, the haplotype sharing among cytotypes was high (Table 5). Out of 49 haplotypes in total, 29 were found to be shared among at least two cytotypes and represented the striking majority of the studied individuals (94.58%). Of the remaining 20 unshared haplotypes, 9 were found only in the tetraploids (representing 2.23% of all individuals). On the contrary, 15 haplotypes (4 shared among the other cytotypes and 11 found in single cytotypes, representing 7.92% of all individuals) were not present in the tetraploids.

### Testing for recurrent allopolyploidisation events involving third species

The AFLP scoring of the 303 individuals of *P. puberula* and its closely related species resulted in 335 polymorphic markers after removal of non-reproducible and non-informative fragments, with an error rate of 1.09% (data accessible as Table S6 in Electr. Suppl. S2). Duplicated genotypes defined by a threshold of seven pairwise distances were excluded from further analyses (Table S6 in Electr. Suppl. S2).

**Fig. 1:**
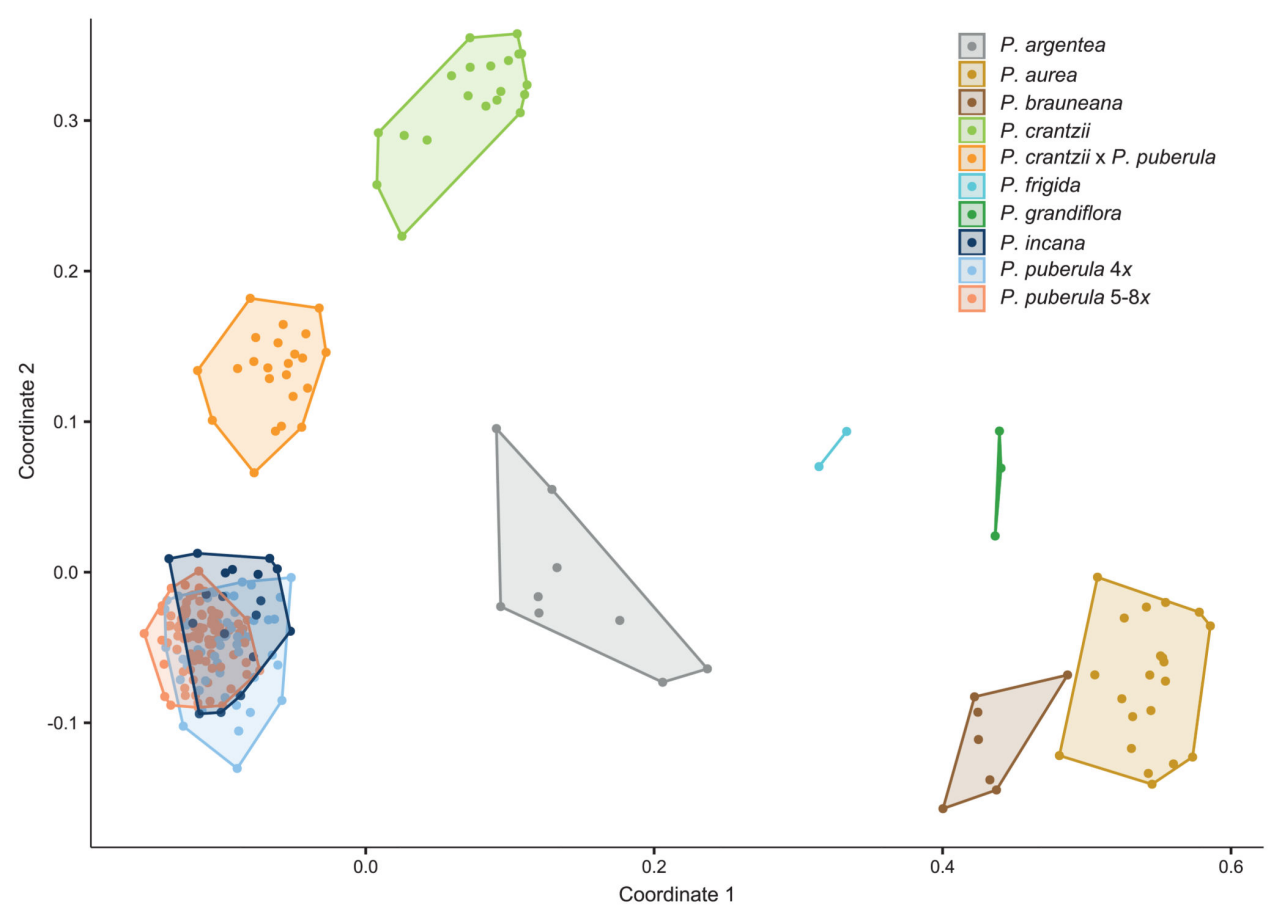
Principal Coordinate Analysis of 251 unique genotypes of nine *Potentilla* L. taxa based on 335 AFLP markers. The coordinates 1 and 2 explain 26.66% and 7.90% of the total genetic variation, respectively.

The interspecific PCoA supported the distinctness of species (Fig. 1), with the first two coordinates explaining 26.66% and 7.90% of the genetic variation, respectively. Only *P. incana* overlapped *P. puberula*, and no distinction was found among tetraploid and penta- to octoploid *P. puberula*. Twenty-three additional unique genotypes originally classified as *P. puberula* formed a separate group intermediate to *P. puberula* and *P. crantzii* consistent with a hybrid origin.

**Fig. 2:**
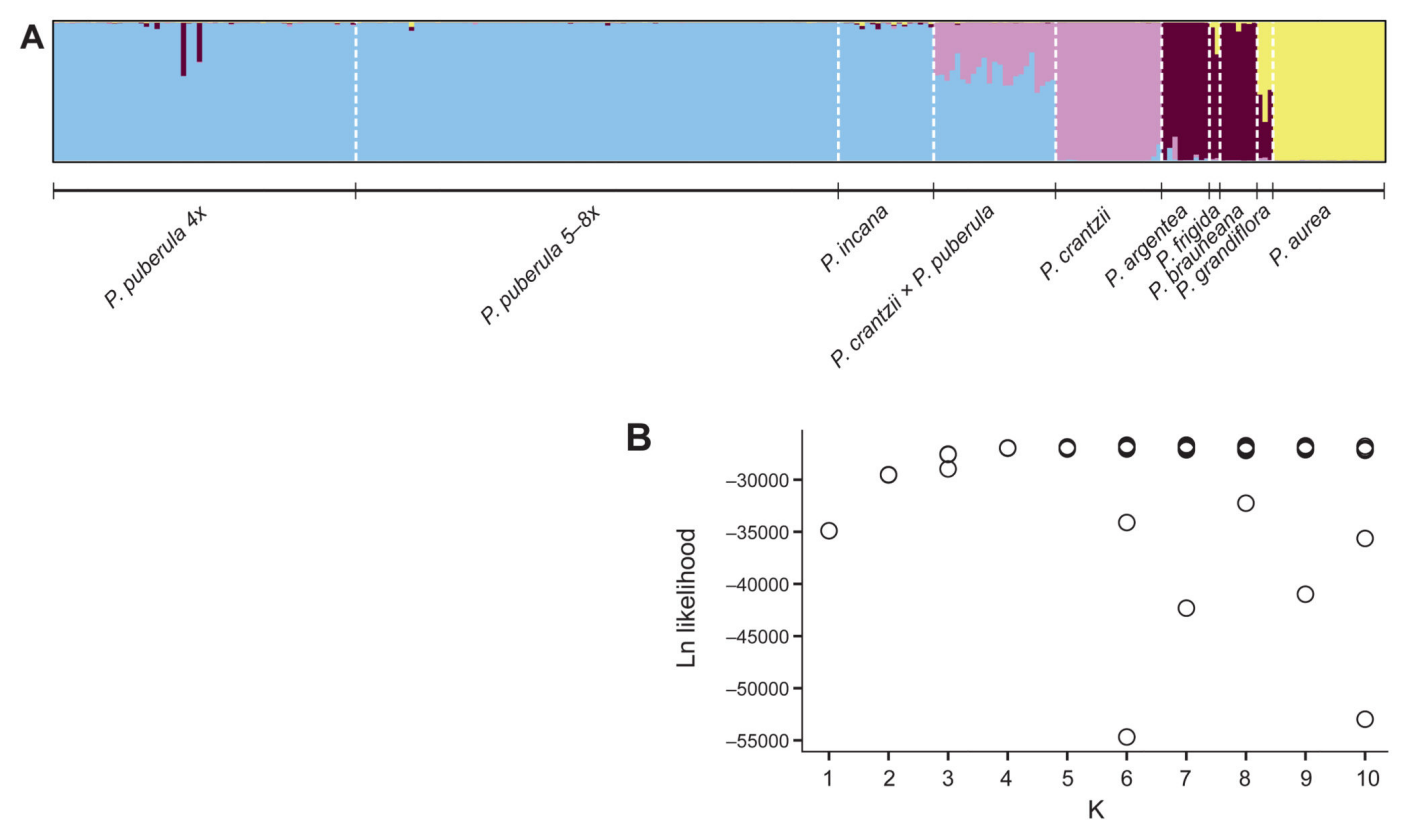
Individual cluster assignment of 251 single genotypes of nine *Potentilla* L. taxa resulting from a STRUCTURE analysis based on 335 AFLP markers. **A,** K = 4, run with the highest likelihood; **B,** Logarithmic likelihood of each run per K value.

The result was confirmed by the interspecific STRUCTURE analysis (Fig. 2). At K = 4, all cytotypes of *P. puberula* as well as *P. incana* were assigned to one cluster, while *P. argentea*, *P. aurea*, and *P. crantzii* were assigned to the remnant three clusters. *Potentilla brauneana*, *P. frigida* and *P. grandiflora* were assigned mainly to the *P. argentea* cluster, although they were assigned to the *P. aurea* cluster in most of the other (less likely) runs (Electr. Suppl. 1: Fig. S3), probably because of the low number of individuals. Two tetraploid individuals of *P. puberula* showed some introgression from a species belonging to the *P. argentea* cluster, but no penta- to octoploid *P. puberula* showed any sign of introgression from other species. The putative hybrids *P. crantzii × P. puberula* were clearly admixed among the two assumed parental species. K > 4 yielded only admixed clusters with individual assignments below 5%.

### Molecular relationships among intraspecific cytotypes and reproductive modes

**Fig. 3:**
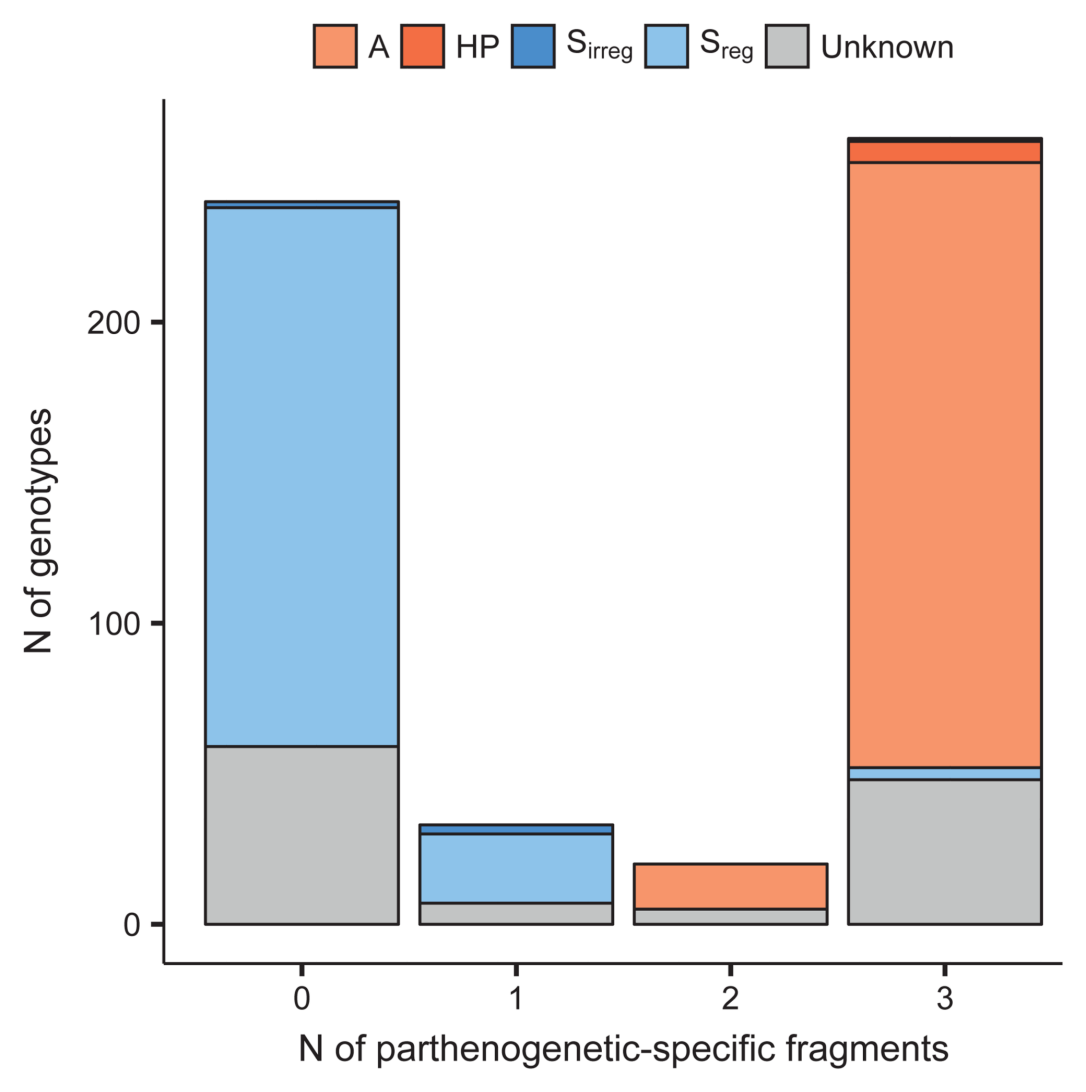
Occurrence of three presumably parthenogenetic-specific AFLP fragments (170 VIC, 219 FAM, 286 FAM) in genotypes of *Potentilla puberula* Krašan classified by reproductive mode. A: apomixis; HP: haploid parthenogenesis; S_irreg_: irregular sexuality (B_III_ hybrids formation); S_reg_: regular sexuality.

In *P. puberula*, three fragments (170 VIC, 219 FAM, 286 FAM) were present at constantly high frequencies in the high-ploidy cytotypes (respectively, 93.31%, 91.64% and 90.30% of genotypes) but low frequencies in the tetraploids (respectively, 3.14%, 1.18% and 8.24%). The fragments tended to co-occur within individuals, as 86.29% of penta- to octoploid genotypes had all three markers, while 89.02% of the tetraploids did not have any of those. Moreover, among 435 genotypes for which reproductive information was available, all genotypes which formed seeds parthenogenetically (independently on the megagametophyte origin) had at least two of the three fragments and 93.27% had all three, whereas 86.89% of genotypes which formed seeds by regular sexuality had none (Fig. 3). Only five high-ploidy genotypes for which only zygotic embryo development was found (corresponding to 1.94% of sexual, 16.67% of B_III_ and 1.67% of high-ploidy genotypes) had all three fragments.

**Fig. 4:**
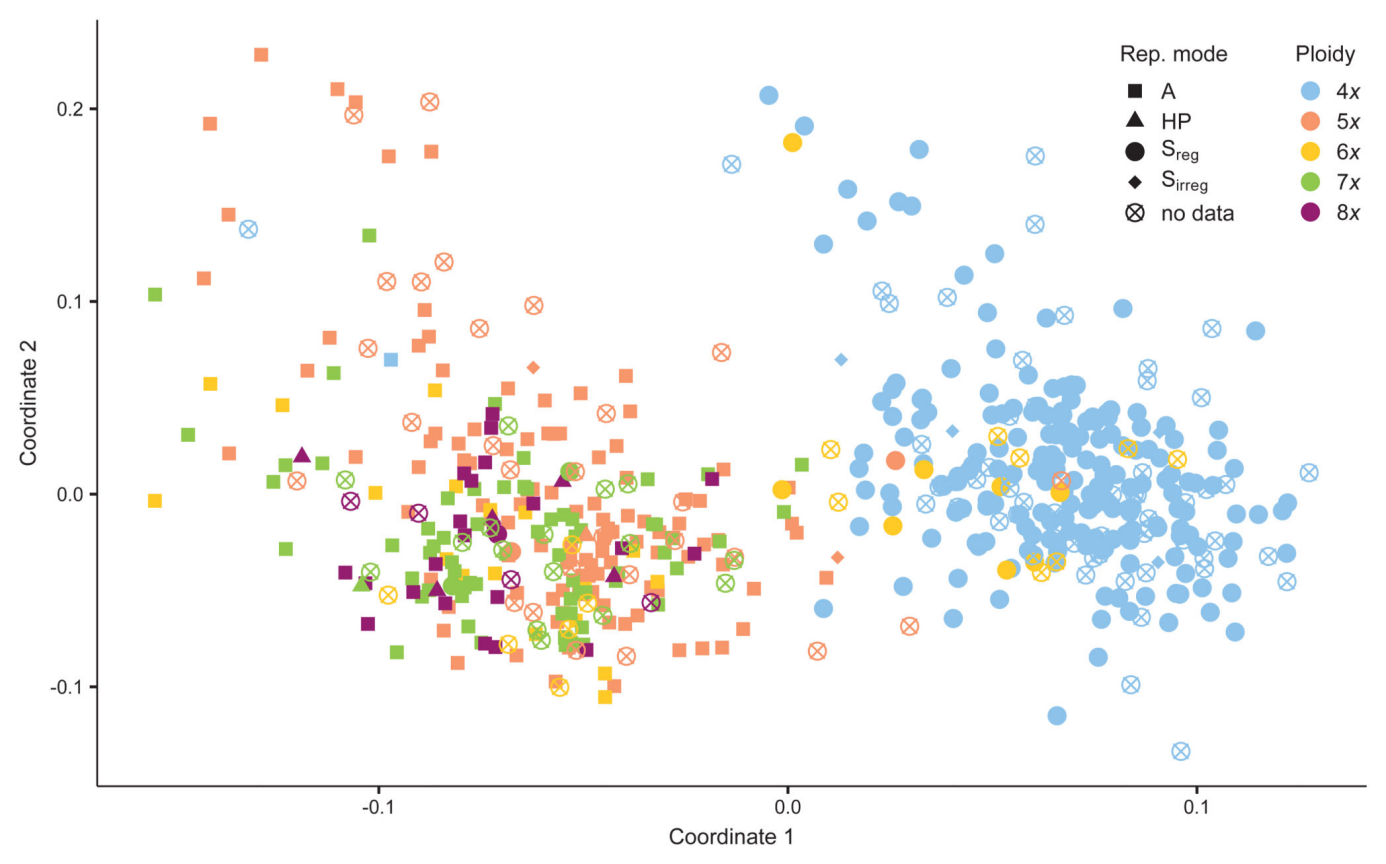
Principal coordinate analysis of 554 unique genotypes of *Potentilla puberula* Krašan based on 370 AFLP markers. The coordinates 1 and 2 explain 4.04% and 2.51% of the total genetic variation, respectively. A: apomixis; HP: haploid parthenogenesis; S_irreg_: irregular sexuality (B_III_ hybrids formation); S_reg_: regular sexuality.

The first two coordinates of the PCoA accounted for only 4.04% and 2.51% of the total variation, respectively (Fig. 4). The first coordinate basically separated the sexual tetraploids from the high-ploidy genotypes. Exceptions are all the hexaploids and two of the pentaploids sampled together with tetraploids in natural populations, which clustered with the first. On the contrary, only two tetraploid genotypes were assigned to the group of the high-ploidy levels. The differentiation was probably mainly due to the three presumably parthenogenetic-specific fragments and largely disappeared when these were removed from the analysis (Electr. Suppl. 1: Fig. S4). None of the highploidy cytotypes formed a distinct subgroup. Rather, cytotypes were fairly mixed in the PCoA.

**Fig. 5:**
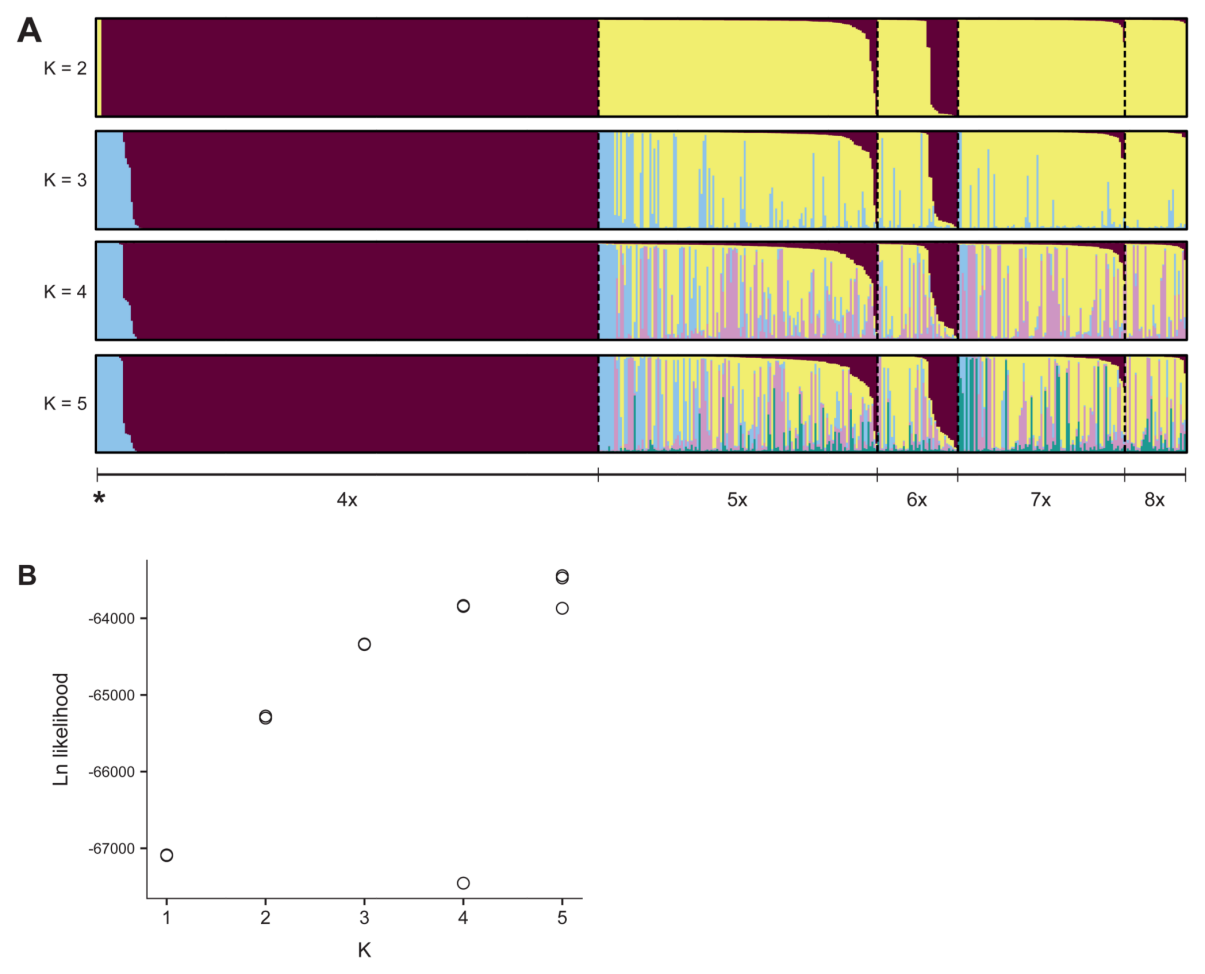
Results of the intraspecific STRUCTURE analysis of 554 single genotypes of *Potentilla puberula* Krašan. **A,** Individual cluster membership based on the run with the highest likelihood per K. The asterisk indicates apomictic tetraploid genotypes. **B,** Logarithmic likelihood of single runs per K value.

At K = 2, the intraspecific STRUCTURE analysis well differentiated the tetraploids and most high-ploidy cytotypes (Fig. 5). Exceptions involved two tetraploid genotypes (one apomictic and one for which no seeds were available) assigned to the high-ploidy cluster and 14 sexual penta- and hexaploids which were assigned to the tetraploid cluster with a membership higher than 90% (Table 3). Among the high-ploidy genotypes, 13 were variously admixed by the tetraploid cluster (Table 3). Most of the other high-ploidy genotypes formed an alternative defined genetic cluster (i.e., with assignation to the tetraploid cluster lower than 10%). At higher values of K, a second tetraploid cluster was found, mostly associated to phylogeographical signal in East Tyrol, and was shared with apomictic individuals from the same geographical area (Fig. 5). At K = 4 and K = 5, additional high-ploidy clusters were obtained, and several genotypes showed admixture among two or more of these clusters (Fig. 5).

Since the second tetraploid cluster found at K > 2 might reflect a different phylogeographical history, rather than representing a real admixture among the tetraploids and the high ploidy genotypes assigned to this group, we ran a STRUCTURE analysis including only the 105 unique genotypes from Eastern Tyrol (Electr. Suppl. 1: Fig. S5). This second analysis resulted in the same pattern evidenced by the general analysis, confirming an effect of geographical structure. At K = 2, the tetraploids were clearly distinct from the penta- to octoploids, with three tetraploids assigned to the high ploidy cluster, while at higher values of K only additional apomictic clusters were found (Electr. Suppl. 1: Fig. S5A).

## Discussion

In this study, we aimed to identify the origin of extant apomictic genotypes of *Potentilla puberula* collected in the field, discriminating in particular among an allopolyploid and an intraspecific origin, and searching for indications for reproductive transfer of apomixis versus its *de novo* origin. Both the PCoA and the intraspecific STRUCTURE analyses evidenced a slight but clear genetic differentiation of sexuals – mostly tetraploids – from apomictic penta- to octoploids (“high ploidy levels”). Main cause of this differentiation is the association of three AFLP alleles with parthenogenetic genotypes (Figs. 3, 4; Electr. Suppl. 1: Fig. S4). In general, our data on neutral genetic variation clearly shows that polyploidisation in *P. puberula* does not involve contributions of other sympatric species, but they allow for drawing conclusions on the ongoing reproductive interactions in this sexual-apomictic system.

### Autopolypoidisation of sexuals did not involve a change in reproductive mode

Although our study did not investigate the intra-individual variation in reproductive modes, we are nevertheless able to draw conclusions at the level of the identified genetic clusters. In particular, no progeny was found to be parthenogenetically derived from the individuals clustering with the sexual group, but sexually derived seeds were found from apomictic penta- to octoploid individuals clustering with the apomictic group. This suggests that the highploidy individuals, mostly hexaploids which clustered with the sexual tetraploids and formed seeds by regular sexuality were derived by spontaneous autopolyploidisation of tetraploids. Auto-hexaploids would originate by sexual fusion of a reduced and an unreduced gamete of tetraploid individuals (i.e., B_III_, sensu Bicknell & al., 2003). The production of unreduced gametes has been observed in *P. puberula*: in an ex-situ crossing experiment, Dobeš & al. (2018) found the occasional production of seeds from unreduced egg cells (1.09%, Table 6) in sexual tetraploids. Even 2.03% of tetraploids seeds measured within our study fall into this category (Table 3). Simple backcrossing of auto-hexaploids with tetraploids might be responsible for the origin of the single exceptional auto-pentaploid we found.

The result is in line with co-occurrence patterns of cytotypes, since the sexual auto-hexaploids mostly occurred in tetraploid populations only. However, frequencies of hexaploids were remarkable (5.00%–9.52%, Appendix 1), an observation which indicates that functional sexuality might allow them to be relatively stable in a population, in spite of minority cytotype disadvantages (Levin, 1975). Vice-versa, irregularities in meiosis and lower chances to originate would make auto-pentaploids rare and ephemeral.

Spontaneous polyploidisation of diploids via fertilisation of unreduced egg cells by reduced pollen was observed in natural populations of *Ranunculus kuepferi* (Schinkel & al., 2017). However, triploids reproduce in this species mostly by apomixis and occur only in the contact zone of sexual diploids and apomictic tetraploids (Schinkel & al., 2016), suggesting an inter-cytotype origin and that they might represent the first step towards the establishment of new apomictic tetraploids (Schinkel & al., 2017). In contrast, no apomictically derived seed was found in auto-hexaploids of *P. puberula* and their absence from apomictic populations suggests that they are not “hexaploid bridges” leading to the formation of apomictic lineages. In conclusion, the evidence of sexuality in autopolyploids does not support the hypothesis that apomixis arises directly via polyploidisation events as nucleotypic effect.

### Cytological pathways to new apomictic lineages involve apomicts

The PCoA, the intraspecific STRUCTURE analysis and the haplotype sharing revealed that none of the apomictic cytotypes represents an independent lineage. On the contrary, the high genetic similarity of apomictic cytoypes strongly supports the hypothesis of frequent changes in ploidy. Overall, we found indication for three principal pathways explaining the origin of newly formed apomicts within *P. puberula*, importantly all involving high ploidy apomicts at least as one parent: self-fertilisation of unreduced egg cells, sexual recombination within apomicts and crosses among apomicts and sexual tetraploids.

Self-fertilisation of unreduced egg cells (i.e., formation of S_III_, sensu Bicknell & al., 2003), is suggested by the finding of sporadic inter-ploidal clonality (Table 4). This particular type of clonality can be explained by fusion of an apomeiotically derived (i.e., unreduced and non-recombined) and a meiotically recombined copy of the same genome. Since dosage of AFLP markers was not diagnosed, this pathway would not result in visible genotyping changes. The ploidy levels of individuals sharing the same genotype in this study mirrored very well this hypothesis: the most frequent cases of genotype sharing were among penta- and hepta-/octoploids (Table 4), in which unreduced pentaploid egg cells might have been fertilised by (irregularly) reduced di- to triploid self pollen. Fertilisation of unreduced egg cells is relatively likely in apomictic *P. puberula*, since it was the most frequent mode of seed formation among the aberrant pathways observed by Dobeš & al. (2018) involving 3.56% of the progeny of the apomicts.

Sexual recombination among apomicts involving both selfing and outcrossing emerged from our data as a major source of genotypic variation within the apomictic group. In the first instance, the intra-population distribution of pairwise genotypic distances among apomicts was not bi-modal as expected in presence of clones and fully recombinant (i.e., derived from outcrossing) genotypes but showed intermediate values (Electr. Suppl. 1: Fig. S2). Although the skewness of the left peak (the clonal distribution) might be also explained with the presence of somatic mutations (as in the case of *Ranunculus carpaticola* Soó, Paun & al., 2006), this cannot account for inter-individual distances intermediate between the two peaks. We hypothesise, thus, that some individuals originated by self-fertilisation involving reduced egg cells (i.e., S_II_, sensu Bicknell & al., 2003), a process which would not induce a change in ploidy, but recombines the maternal apomictic genotype. The result is in line with Dobeš & al. (2018), who found that the ploidy of apomictic mothers is often retrieved in sexually derived progeny of high-ploidy individuals (2.70% of the progeny). Penta- to octoploid *P. puberula* is self-compatible and selfing was frequent despite application of cross-pollen on the stigmata of non-emasculated flowers (Dobeš & al., 2013b), which suggests that selfing might play a relevant role in the origin of new genotypes.

A second evidence for sexual genetic recombination within the apomictic ploidy levels comes from the admixture analysis, which partially assigned several individuals to different apomictic clusters. The absence of cytotype-specific genetic clusters makes impossible to reveal the concrete cytological pathways giving rise to new genotypes in terms of ploidy of involved parents and gametes. However, the overall high genetic similarity of the different apomictic cytotypes strongly supports the hypothesis of frequent heteroploid crosses resulting in the formation of new cytotypes. Although meiosis in odd-ploids is expected to be irregular, female reduced gametes are nevertheless produced side by side with unreduced ones and both types of gametes can be fertilised by heteroploid pollen, also as a result of the tolerance of unbalanced genomic ratios in the endosperm (Dobeš & al., 2018). The large cpDNA haplotype sharing among apomictic cytotypes (regardless of the sharing with tetraploids) gives additional support to this scenario. Data obtained by Dobeš & al. (2018) indicate that, although most of apomicts reproduce sexually only at a low rate (3.03 % of the measured seeds was sexually derived), sexuality is maintained at significant rates (20.38% of the seeds) in some facultative apomicts (Table 6), which might play a key role for maintenance of the genetic variability of the population and the formation of new genotypes within the apomictic group.

Heteroploid crosses involving sexual tetraploids and apomictic penta- to octoploids were revealed by the admixture analyses: some penta- to heptaploid individuals were at least partially admixed with the sexual tetraploid cluster. Changes in ploidy in the progeny of tetraploids observed within heteroploid crossing experiments (Dobeš & al., 2018), showed that penta- and hexaploid embryos are usually produced when high-ploidy individuals fertilise tetraploids, although up to octoploid embryos may be formed in case of unreduced egg cells. No unreduced pollen has been found to successfully fertilise tetraploids, probably because of unsustainable genomic imbalance (Dobeš & al., 2018). However, successful cross-fertilisation of tetraploids and high ploidy individuals appears uncommon in wild populations, because of the low frequency of genotypes genetically admixed between the tetra- and the high ploidy genetic cluster (Fig. 5). Ex-situ heteroploid crossing experiments with tetraploids as mothers revealed that viable out-crossing-derived seeds could be produced, but the resulting seed set was significantly lower compared to homoploid tetraploid crosses (Dobeš & al., 2018). In addition, fertilisation of sexuals by apomicts was nearly non-existent in reproductively mixed populations in their natural habitats, an observation explained by activity of prezygotic crossing barriers (Dobeš & al., 2013b). Our results, thus, are in line with the existence of a reproductive, although incomplete, barrier among tetraploids and penta- to octoploids in natural populations. Interestingly, all except one individual which showed 10%–90% of assignation to the sexual cluster in the STRUCTURE analysis were sampled from populations in which tetra- and penta- to octoploids co-occurred. This confirms the importance of sympatry in a strict sense in shaping the relationships of sexuals with apomicts and supports the idea of possible progressive replacement of sexuals by apomicts via heteroploid crosses. Such process would be delayed by the evolution of reproductive barriers among the two groups (Mogie, 1992; Joshi & Moody, 1995, 1998). In addition, selection against non-functional apomicts arising from the meiotic and sexual recombination of their genomes resulting from hybridisation, may explain the low level of admixture by sexuals, and in turn maintenance of the slight but significant differentiation among reproductive modes.

Although the ongoing heteroploid origin of apomicts involving sexual tetraploids has not been highly relevant in the last generations, the large cpDNA haplotype sharing of tetraploids with penta- to octoploids suggests that recombination events have occurred in historic times extensively and possibly constantly. Spatial and reproductive contact among sexuals and apomicts might have been even more frequent in different ecological conditions, compared to the recent situation (Hülber & al., 2013). The effect of sympatry might have been amplified in glacial refugia, during the postglacial expansion of dry grasslands, fragmentations after postglacial forestation and grassland expansion due to human land-use. Certain situations might have favoured the high-ploidy apomicts as they are expected to have an advantage in environmental conditions at the margin of the ecological range of a certain taxon and in a colonisation phase, due to higher and faster seed production, and to independence from pollinators and mating partners (Hörandl, 2006). It is therefore likely that the apomictic trait has been positively selected in such environmental conditions (Mogie, 1992) and that the relative frequencies of sexuals and apomicts might have been different from nowadays, influencing the rates of heteroploid reproductive success. The cpDNA variation would thus be the result of past admixture which is no more visible at the nuclear level, because of mutations and the confounding effect of sexuality within the apomictic group.

### Apomictic genotypes did not originate by recurrent interspecific hybridisation

Among the studied species, we found substantial rates of hybridisation of *P. puberula* only with *P. crantzii*. With all other related and sympatric taxa, no sign of hybridisation was found. Only a tetraploid population of *P. puberula* (population 95) showed introgression from a taxon among or related to *P. argentea*, *P. braunenana*, *P. frigida* and *P. grandiflora.* The genetic proximity of the largely allopatric *P. incana* to *P. puberula* reflects their very close relatedness. The two species both belong to the *P. verna* agg., a complex of cryptic species (Wolf, 1908; Ehrendorfer, 1973; Kurtto & al., 2004), therefore our AFLP markers probably did not have the resolution to discriminate them.

*Potentilla crantzii* hybridised frequently (in 14 populations) with *P. puberula*. However, the *P. crantzii* × *P. puberula* hybrids were genetically clearly distinct (probably mainly F_1_ hybrids) and we found no relevant introgression from *P. crantzii* in *P. puberula*. Moreover, neither *P. crantzii* nor the other sympatric species presented the three loci possibly associated with apomixis (170 VIC, 219 FAM, 286 FAM), with the exception of two genotypes of *P. crantzii* (from population 224) sampled together with *P. puberula* (population 69).

Based on this study, we can exclude recent recurrent allopolyploid origins of apomictic *P. puberula*, which we confirm as a genetically variable but coherent species (Wolf, 1908; Ehrendorfer, 1973; Paule & al., 2012). Nevertheless, we cannot fully exclude that apomixis was originally historically introduced into *P. puberula* after introgression and gene capture – or alternatively as direct effect of allopolyploidisation (Carman, 1997) – from another now extinct or not sampled species from outside the study area, as the case in other systems (e.g. De Wet & Harlan, 1970; Roche & al., 1999; Sharbel & MitchellOlds, 2001).

### Final remarks

The sexual-apomictic species *Potentilla puberula* offers opportunities to study the relationships and interactions of reproductively differentiated cytotypes. Sympatry and reproductively compatible cytotypes may have drastic effects on the maintenance of obligate sexual populations and culminate in their displacement. However, other factors, such as ecological differentiation or the evolution of stronger reproductive barriers might allow for coexistence of the two groups. Phylogeographical investigations on the study area, together with estimation of the age of the apomicts might help to investigate whether sexual displacement partially already occurred in some populations.

Additionally, the discovery of three parthenogenetic-specific AFLP fragments might lay the foundations for genetic mapping studies on apomixis which are still underrepresented in the Rosaceae family (Ozias-Akins & Van Dijk, 2007). Such studies, together with careful investigation of inheritance of apomixis and its components, are necessary to shed light on the genetics of apomixis in this species and, consequently, to understand the roles of recombination and outcrossing in the genesis of apomicts.

## Supplementary Material

Electronic Supplement 1 (Tables S1, S2; Figs. S1–S5) and Electronic Supplement 2 (MS Excel file with Tables S3–S6) are available from https://doi.org/10.12705/676.8.S1 and https://doi.org/10.12705/676.8.S2, respectively.

Electronic Supplement 1

Electronic Supplement 2

Appendix

## Figures and Tables

**Table 1:** Ploidies and associated reproductive modes in *Potentilla* L. species studied.

Species	Ploidy	Reproductive mode	References
*P. argentea* L.	2*x*	Sexual	Paule & al. (2011); Dobeš & al. (2015)
	6*x*	Apomictic	Paule & al. (2011); Dobeš & al. (2015)
*P. aurea* L.	2*x*	Sexual	Dobeš & al. (2015)
*P. brauneana* Hoppe	2*x*	Likely sexual	–
*P. crantzii* (Crantz) Beck ex Fritsch	4*x*	Sexual	Czapik (1961, 1962)
	5–9*x*	Apomictic	Smith (1963a, b)
*P. frigida* Vill.	4*x*	Sexual	Dobeš & al. (2015)
*P. grandiflora* L.	4*x*	Sexual	Dobeš & al. (2015)
*P. incana* G.Gaertn. & al.	4*x*	Sexual	Dobeš & al. (2015)
*P. puberula* Krašan	4*x*	Sexual	Dobeš & al. (2013b)
	5–8*x*	Apomictic	Dobeš & al. (2013b)

**Table 2:** Reproductive pathways of seed formation and their inference.

Embryo development	Megagametophyte formation	
	Meiosis	Apomeiosis
Fertilisation	Regular sexuality	Irregular sexuality (B_III_ hybrids formation)
	P_♀_ = 1/2 M	P_♀_ = M
	pi < 1.9	pi < 1.9

Parthenogenesis	Haploid parthenogenesis	Apomixis
	P_♀_ = 1/2 P	P_♀_ = P
	pi > 2.1	pi > 2.1

M: ploidy of the mother plant; P_♀_: ploidy of the megagametophyte; pi: peak index.

**Table 3:** Association among genetic clusters as defined by a trained STRUCTURE analysis (see text and Fig. 5), ploidy and reproductive mode of seed formation (N = 491^a^) inferred by FCSS in *Potentilla puberula* Krašan.

Genetic cluster^b^	N individuals	N genotypes	N seeds	A	HP	S_reg_	S_irreg_
Cluster 1 (“Tetraploids”)							
4*x*	253	253	95 (236)	0 (0)	0 (0)	191 (231)	4 (5)
5*x*	1	1	0 (0)	0 (0)	0 (0)	0 (0)	0 (0)
6*x*	13	13	15 (15)	0 (0)	0 (0)	15 (15)	0 (0)

Admixed							
5*x*	9	9	10 (10)	4 (4)	0 (0)	3 (3)	3 (3)
6*x*	3	3	1 (1)	0 (0)	0 (0)	1 (1)	0 (0)
7*x*	1	1	1 (1)	1 (1)	0 (0)	0 (0)	0 (0)

Cluster 2 (“High ploidy”)							
4*x*	3	2	2 (2)	1 (1)	0 (0)	1 (1)	0 (0)
5*x*	225	132	124 (192)	119 (185)	2 (2)	2 (2)	1 (3)
6*x*	42	25	20 (67)	19 (63)	0 (1)	1 (2)	0 (1)
7*x*	131	84	89 (94)	79 (83)	1 (1)	7 (8)	2 (2)
8*x*	45	31	34 (46)^a^	25 (37)^a^	7 (7)^a^	2 (2)	0 (0)

Number of seeds for each reproductive pathway are reported: A: apomixis (apomeiosis and parthenogenesis); HP: haploid parthenogenesis; S_reg_: regular sexuality (female meiosis and fertilisation); S_irreg_: irregular sexuality (B_III_ hybrids formation). Numbers out of parentheses refer to measurements within this study only. Numbers in parentheses include both seeds measured within this study and seeds measured by Dobeš & al. (2018)

a Ten additional seeds produced by octoploid individuals were found to be parthenogenetically derived, but the exact reproductive pathway (i.e., apomixis vs. haploid parthenogenesis) could not be determined.

b Genetic cluster as resulted by the intraspecific STRUCTURE analysis for K = 2. Genotypes were assigned to the two clusters (Cluster 1 “Tetraploids” and Cluster 2 “High ploidy”) when their estimated membership to the pre-defined tetraploid genetic population was respectively over 90% and below 10%. Genotypes were considered admixed among the two clusters when their assignation to Cluster 1 (“Tetraploids”) was over 10% and below 90%.

**Table 4:** AFLP genotypes (defined by a threshold of 12 mismatches) shared among cytotypes of *Potentilla puberula* Krašan. Number of clones are reported by cytotype. The shared genotypes present 2.71% of the genotypes observed.

Genotype	4*x*	5*x*	6*x*	7*x*	8*x*	N clones	N cytotypes sharing genotype
g027	–	1	–	1	–	2	2
g065	–	1	–	3	–	4	2
g100	–	3	–	1	–	4	2
g109	–	2	–	1	–	3	2
g182	–	1	–	1	–	2	2
g198	–	1	–	–	1	2	2
g256	–	–	–	2	1	3	2
g262	1	3	–	1	–	5	3
g302	–	–	2	–	1	3	2
g321	–	1	–	2	–	3	2
g365	–	1	–	–	2	3	2
g389	–	–	1	–	3	4	2
g488	–	3	–	2	–	5	2
g512	–	–	1	–	4	5	2
g527	–	1	1	–	–	2	2

**Table 5:** Number of individuals of *Potentilla puberula* Krašan sharing the same cpDNA haplotype, reported by ploidy level.

Haplotype	4*x*	5*x*	6*x*	7*x*	8*x*	N	N_c_
H18	82	91	18	58	24	273	5
H01	38	38	–	10	7	93	4
H19	24	22	2	1	6	55	5
H17	18	11	1	2	–	32	4
H02	7	2	19	1	–	29	4
H03	12	3	–	7	–	22	3
H31	8	–	–	11	–	19	2
H28	10	–	2	2	–	14	3
H30	3	5	1	–	4	13	4
H29	–	9	–	2	–	11	2
H42	1	7	–	3	–	11	3
H23	3	4	1	2	–	10	4
H26	–	5	–	5	–	10	2
H04	2	2	4	–	–	8	3
H14	–	7	–	1	–	8	2
H08	2	–	3	–	2	7	3
H21	2	5	–	–	–	7	2
H33	1	–	4	2	–	7	3
H41	1	4	–	2	–	7	3
H06	1	1	–	4	–	6	3
H15	5	–	1	–	–	6	2
H37	5	–	1	–	–	6	2
H22	4	1	–	–	–	5	2
H25	–	2	–	1	2	5	3
H32	2	1	1	–	1	5	4
H40	5	–	–	–	–	5	1
H07	–	4	–	–	–	4	1
H24	3	1	–	–	–	4	2
H34	–	4	–	–	–	4	1
H39	–	–	–	4	–	4	1
H27	1	–	–	2	–	3	2
H48	–	–	–	3	–	3	1
H10	2	–	–	–	–	2	1
H16	2	–	–	–	–	2	1
H20	1	–	–	1	–	2	2
H35	–	2	–	–	–	2	1
H38	2	–	–	–	–	2	1
H47	1	–	–	1	–	2	2
H05	–	–	–	–	1	1	1
H09	–	–	1	–	–	1	1
H11	1	–	–	–	–	1	1
H12	–	1	–	–	–	1	1
H13	1	–	–	–	–	1	1
H36	–	–	1	–	–	1	1
H43	1	–	–	–	–	1	1
H44	–	–	1	–	–	1	1
H45	–	1	–	–	–	1	1
H46	1	–	–	–	–	1	1
H49	1	–	–	–	–	1	1

N: total number of individuals per haplotype; N_c_: number of cytotypes sharing the same haplotype.

**Table 6:** Percentages of seeds derived by different reproductive modes in 37 prevalently apomictic, 9 facultative apomictic, and 12 obligate sexual individuals.

Individual reproductive mode	A	HP	S_reg_	S_irreg_	N seeds
Apomictic^a^	96.87	0.10	0.78	2.25	1023
Mixed	75.83	3.79	12.32	8.06	211
Sexual^b^	0.00	0.00	98.91	1.09	276

Data derived from Dobeš & al. (2018). A: apomixis (apomeiosis and parthenogenesis); HP: haploid parthenogenesis; Sreg: regular sexuality (meiosis and fertilisation); Sirreg: irregular sexuality (BIII hybrids formation).

a All cytotypes represented.

b Only tetraploid individuals were determined as obligate sexual.
