# Supplementary material for: Sexual intraspecific recombination but not de novo origin governs the genesis of new apomictic genotypes in Potentilla puberula (Rosaceae)
Source: Taxon. Author manuscript; Available in PMC 2019 Feb 20. (PMC6382066)
Supplement: Electronic Supplement 1 [file NIHMS81441-supplement-Electronic_Supplement_1.pdf]

Vol. 67 (6) • December 2018

TAXON

International Journal of Taxonomy, Phylogeny and Evolution

Electronic Supplement 1 to

Sexual intraspecific recombination but not *de novo* origin governs the genesis of new apomictic genotypes in *Potentilla puberula* (Rosaceae)

Flavia Domizia Nardi, Christoph Dobeš, Dorothee Müller, Tobias Grasegger, Tuuli Myllynen, Henar Alonso-Marcos & Andreas Tribsch

Taxon 67: 1108–1131 (https://doi.org/10.12705/676.8)

https://doi.org/10.12705/676.8.S1 (Electronic Supplement 2: https://doi.org/10.12705/676.8.S2)

Table S1. Herbarium vouchers representing studied taxa and deposited in W and GOET.

Taxon	Population	Country	Province	Original collectors	Collector ex cult.	Collection number	Herbarium
Potentilla argentea L.	195	Italy	Trentino-Alto Adige	*Tribsch, A., Nardi, F.D.*	*Nardi, F.D.*	*Nardi, F.D. 195_03*	(W 2018-0002034)
Potentilla argentea L.	195	Italy	Trentino-Alto Adige	*Tribsch, A., Nardi, F.D.*	*Nardi, F.D.*	*Nardi, F.D. 195_04*	(W 2018-0002036)
Potentilla argentea L.	195	Italy	Trentino-Alto Adige	*Tribsch, A., Nardi, F.D.*	*Nardi, F.D.*	*Nardi, F.D. 195_05*	(W 2018-0002033)
Potentilla argentea L.	198	Italy	Trentino-Alto Adige	*Alonso-Marcos, H., Nardi, F.D.*	*Nardi, F.D.*	*Nardi, F.D. 198_08*	(W 2018-0002032)
Potentilla argentea L.	198	Italy	Trentino-Alto Adige	*Alonso-Marcos, H., Nardi, F.D.*	*Nardi, F.D.*	*Nardi, F.D. 198_10*	(W 2018-0002031)
Potentilla aurea L.	194	Italy	Trentino-Alto Adige	*Alonso-Marcos, H., Nardi, F.D.*	*Nardi, F.D.*	*Nardi, F.D. 194_08*	(W 2018-0002038)
Potentilla aurea L.	199	Italy	Trentino-Alto Adige	*Alonso-Marcos, H., Nardi, F.D.*	*Nardi, F.D.*	*Nardi, F.D. 199_04*	(W 2018-0002035)
Potentilla brauneana Hoppe	205	Austria	Tyrol	*Hörandl, E., Hörandl, S., Hadacek, F.*		*Hörandl, E., Hörandl, S., Hadacek, F. 10247*	(GOET)
Potentilla brauneana Hoppe	206	Italy	Trentino-Alto Adige	*Hörandl, E., Hörandl, S., Hadacek, F.*		*Hörandl, E., Hörandl, S., Hadacek, F. 10267*	(GOET)
Potentilla crantzii (Crantz) Beck ex Fritsch	207	Italy	Trentino-Alto Adige	*Hörandl, E., Hörandl, S., Hadacek, F.*		*Hörandl, E., Hörandl, S., Hadacek, F. 10266*	(GOET)
Potentilla crantzii (Crantz) Beck ex Fritsch	208	Switzerland	Grisons	*Hörandl, E., Hörandl, S., Hadacek, F.*		*Hörandl, E., Hörandl, S., Hadacek, F. 10278*	(GOET)
Potentilla crantzii (Crantz) Beck ex Fritsch × *P. puberula* Krašan	026	Austria	Tyrol	*Stifter, S., Haider, J.A.*	*Nardi, F.D.*	*Nardi, F.D. 026_31*	(W 2018-0002240)
Potentilla crantzii (Crantz) Beck ex Fritsch × *P. puberula* Krašan	065	Italy	Trentino-Alto Adige	*Alonso-Marcos, H., Dobeš, C.*	*Nardi, F.D.*	*Nardi, F.D. 065_12*	(W 2018-0002224)
Potentilla crantzii (Crantz) Beck ex Fritsch × *P. puberula* Krašan	069	Italy	Trentino-Alto Adige	*Tribsch, A., Nardi, F.D.*	*Nardi, F.D.*	*Nardi, F.D. 069_02*	(W 2018-0002214)
Potentilla crantzii (Crantz) Beck ex Fritsch × *P. puberula* Krašan	069	Italy	Trentino-Alto Adige	*Tribsch, A., Nardi, F.D.*	*Nardi, F.D.*	*Nardi, F.D. 069_03*	(W 2018-0002247)
Potentilla crantzii (Crantz) Beck ex Fritsch × *P. puberula* Krašan	098	Italy	Trentino-Alto Adige	*Alonso-Marcos, H., Nardi, F.D.*	*Nardi, F.D.*	*Nardi, F.D. 098_01*	(W 2018-0002148)
Potentilla crantzii (Crantz) Beck ex Fritsch × *P. puberula* Krašan	104	Italy	Trentino-Alto Adige	*Alonso-Marcos, H., Nardi, F.D.*	*Nardi, F.D.*	*Nardi, F.D. 104_07*	(W 2018-0002167)

Table S1. Herbarium vouchers representing studied taxa and deposited in W and GOET.

Taxon	Population	Country	Province	Original collectors	Collector ex cult.	Collection number	Herbarium
Potentilla crantzii (Crantz) Beck ex Fritsch × *P. puberula* Krašan	139	Austria	Tyrol	*Alonso-Marcos, H., Nardi, F.D.*	*Nardi, F.D.*	*Nardi, F.D. 139_01*	(W 2018-0002125)
Potentilla crantzii (Crantz) Beck ex Fritsch × *P. puberula* Krašan	147	Italy	Trentino-Alto Adige	*Stifter, S., Haider, J.A.*	*Nardi, F.D.*	*Nardi, F.D. 147_07*	(W 2018-0002075)
Potentilla crantzii (Crantz) Beck ex Fritsch × *P. puberula* Krašan	147	Italy	Trentino-Alto Adige	*Stifter, S., Haider, J.A.*	*Nardi, F.D.*	*Nardi, F.D. 147_15*	(W 2018-0002116)
Potentilla crantzii (Crantz) Beck ex Fritsch × *P. puberula* Krašan	150	Italy	Trentino-Alto Adige	*Stifter, S., Haider, J.A.*	*Nardi, F.D.*	*Nardi, F.D. 150_17*	(W 2018-0002112)
Potentilla crantzii (Crantz) Beck ex Fritsch × *P. puberula* Krašan	151	Italy	Trentino-Alto Adige	*Stifter, S., Haider, J.A.*	*Nardi, F.D.*	*Nardi, F.D. 151_13*	(W 2018-0002109)
Potentilla crantzii (Crantz) Beck ex Fritsch × *P. puberula* Krašan	174	Italy	Friuli-Venezia Giulia	*Dobeš, C.*	*Nardi, F.D.*	*Nardi, F.D. 174_07*	(W 2018-0002084)
Potentilla crantzii (Crantz) Beck ex Fritsch × *P. puberula* Krašan	184	Italy	Friuli-Venezia Giulia	*Dobeš, C.*	*Nardi, F.D.*	*Nardi, F.D. 184_01*	(W 2018-0002076)
Potentilla frigida Vill.	209	Switzerland	Grisons	*Hörandl, E., Hörandl, S., Hadacek, F.*		*Hörandl, E., Hörandl, S., Hadacek, F. 10277*	(GOET)
Potentilla frigida Vill.	210	Italy	Trentino-Alto Adige	*Hörandl, E., Hörandl, S., Hadacek, F.*		*Hörandl, E., Hörandl, S., Hadacek, F. 10283*	(GOET)
Potentilla incana G.Gaertn., B.Mey. & Scherb.	197	Italy	Lombardy	*Alonso-Marcos, H., Nardi, F.D., Dobeš, C.*	*Nardi, F.D.*	*Nardi, F.D. 197_06*	(W 2018-0002030)
Potentilla incana G.Gaertn., B.Mey. & Scherb.	197	Italy	Lombardy	*Alonso-Marcos, H., Nardi, F.D., Dobeš, C.*	*Nardi, F.D.*	*Nardi, F.D. 197_10*	(W 2018-0002029)
Potentilla puberula Krašan	005	Austria	Tyrol	*Stifter, S., Haider, J.A.*	*Nardi, F.D.*	*Nardi, F.D. 005_54*	(W 2018-0002246)
Potentilla puberula Krašan	006	Austria	Tyrol	*Stifter, S., Haider, J.A.*	*Nardi, F.D.*	*Nardi, F.D. 006_54*	(W 2018-0002245)
Potentilla puberula Krašan	006	Austria	Tyrol	*Stifter, S., Haider, J.A.*	*Nardi, F.D.*	*Nardi, F.D. 006_55*	(W 2018-0002244)
Potentilla puberula Krašan	007	Austria	Tyrol	*Stifter, S., Haider, J.A.*	*Nardi, F.D.*	*Nardi, F.D. 007_54*	(W 2018-0002243)
Potentilla puberula Krašan	007	Austria	Tyrol	*Stifter, S., Haider, J.A.*	*Nardi, F.D.*	*Nardi, F.D. 007_62*	(W 2018-0002242)
Potentilla puberula Krašan	016	Austria	Tyrol	*Stifter, S., Haider, J.A.*	*Nardi, F.D.*	*Nardi, F.D. 016_52*	(W 2018-0002241)

Table S1. Herbarium vouchers representing studied taxa and deposited in W and GOET.

Taxon	Population	Country	Province	Original collectors	Collector ex cult.	Collection number	Herbarium
Potentilla puberula Krašan	022	Austria	Tyrol	*Stifter, S., Haider, J.A.*	*Nardi, F.D.*	*Nardi, F.D. 022_60*	(W 2018-0002236)
Potentilla puberula Krašan	026	Austria	Tyrol	*Stifter, S., Haider, J.A.*	*Nardi, F.D.*	*Nardi, F.D. 026_42*	(W 2018-0002222)
Potentilla puberula Krašan	029	Austria	Tyrol	*Stifter, S., Haider, J.A.*	*Nardi, F.D.*	*Nardi, F.D. 029_44*	(W 2018-0002239)
Potentilla puberula Krašan	029	Austria	Tyrol	*Stifter, S., Haider, J.A.*	*Nardi, F.D.*	*Nardi, F.D. 029_47*	(W 2018-0002237)
Potentilla puberula Krašan	029	Austria	Tyrol	*Stifter, S., Haider, J.A.*	*Nardi, F.D.*	*Nardi, F.D. 029_48*	(W 2018-0002238)
Potentilla puberula Krašan	031	Austria	Tyrol	*Stifter, S., Haider, J.A.*	*Nardi, F.D.*	*Nardi, F.D. 031_41*	(W 2018-0002235)
Potentilla puberula Krašan	039	Austria	Tyrol	*Stifter, S., Haider, J.A.*	*Nardi, F.D.*	*Nardi, F.D. 039_45*	(W 2018-0002233)
Potentilla puberula Krašan	040	Austria	Tyrol	*Stifter, S., Haider, J.A.*	*Nardi, F.D.*	*Nardi, F.D. 040_46*	(W 2018-0002234)
Potentilla puberula Krašan	040	Austria	Tyrol	*Stifter, S., Haider, J.A.*	*Nardi, F.D.*	*Nardi, F.D. 040_52*	(W 2018-0002232)
Potentilla puberula Krašan	043	Austria	Tyrol	*Stifter, S., Haider, J.A.*	*Nardi, F.D.*	*Nardi, F.D. 043_24*	(W 2018-0002231)
Potentilla puberula Krašan	045	Austria	Tyrol	*Stifter, S., Haider, J.A.*	*Nardi, F.D.*	*Nardi, F.D. 045_34*	(W 2018-0002230)
Potentilla puberula Krašan	054	Austria	Tyrol	*Stifter, S., Haider, J.A.*	*Nardi, F.D.*	*Nardi, F.D. 054_19*	(W 2018-0002221)
Potentilla puberula Krašan	061	Italy	Trentino-Alto Adige	*Alonso-Marcos, H., Nardi, F.D.*	*Nardi, F.D.*	*Nardi, F.D. 061_07*	(W 2018-0002229)
Potentilla puberula Krašan	062	Italy	Veneto	*Alonso-Marcos, H., Nardi, F.D., Tribsch, A., Dobeš, C.*	*Nardi, F.D.*	*Nardi, F.D. 062_06*	(W 2018-0002228)
Potentilla puberula Krašan	063	Italy	Veneto	*Alonso-Marcos, H., Nardi, F.D., Tribsch, A., Dobeš, C.*	*Nardi, F.D.*	*Nardi, F.D. 063_06*	(W 2018-0002227)
Potentilla puberula Krašan	064	Italy	Veneto	*Alonso-Marcos, H., Nardi, F.D., Tribsch, A., Dobeš, C.*	*Nardi, F.D.*	*Nardi, F.D. 064_01*	(W 2018-0002223)
Potentilla puberula Krašan	064	Italy	Veneto	*Alonso-Marcos, H., Nardi, F.D., Tribsch, A., Dobeš, C.*	*Nardi, F.D.*	*Nardi, F.D. 064_09*	(W 2018-0002220)
Potentilla puberula Krašan	065	Italy	Trentino-Alto Adige	*Alonso-Marcos, H., Dobeš, C.*	*Nardi, F.D.*	*Nardi, F.D. 065_04*	(W 2018-0002225)
Potentilla puberula Krašan	065	Italy	Trentino-Alto Adige	*Alonso-Marcos, H., Dobeš, C.*	*Nardi, F.D.*	*Nardi, F.D. 065_07*	(W 2018-0002226)
Potentilla puberula Krašan	066	Italy	Trentino-Alto Adige	*Alonso-Marcos, H., Dobeš, C.*	*Nardi, F.D.*	*Nardi, F.D. 066_01*	(W 2018-0002217)
Potentilla puberula Krašan	066	Italy	Trentino-Alto Adige	*Alonso-Marcos, H., Dobeš, C.*	*Nardi, F.D.*	*Nardi, F.D. 066_06*	(W 2018-0002219)
Potentilla puberula Krašan	066	Italy	Trentino-Alto Adige	*Alonso-Marcos, H., Dobeš, C.*	*Nardi, F.D.*	*Nardi, F.D. 066_09*	(W 2018-0002218)
Potentilla puberula Krašan	067	Italy	Trentino-Alto Adige	*Alonso-Marcos, H., Dobeš, C.*	*Nardi, F.D.*	*Nardi, F.D. 067_01*	(W 2018-0002216)
Potentilla puberula Krašan	067	Italy	Trentino-Alto Adige	*Alonso-Marcos, H., Dobeš, C.*	*Nardi, F.D.*	*Nardi, F.D. 067_09*	(W 2018-0002215)

Table S1. Herbarium vouchers representing studied taxa and deposited in W and GOET.

Taxon	Population	Country	Province	Original collectors	Collector ex cult.	Collection number	Herbarium
Potentilla puberula Krašan	068	Italy	Lombardy	*Alonso-Marcos, H., Dobeš, C.*	*Nardi, F.D.*	*Nardi, F.D. 068_15*	(W 2018-0002213)
Potentilla puberula Krašan	070	Italy	Trentino-Alto Adige	*Tribsch, A., Nardi, F.D.*	*Nardi, F.D.*	*Nardi, F.D. 070_09*	(W 2018-0002212)
Potentilla puberula Krašan	071	Italy	Lombardy	*Alonso-Marcos, H., Nardi, F.D., Dobeš, C.*	*Nardi, F.D.*	*Nardi, F.D. 071_04*	(W 2018-0002048)
Potentilla puberula Krašan	072	Italy	Lombardy	*Alonso-Marcos, H., Nardi, F.D., Dobeš, C.*	*Nardi, F.D.*	*Nardi, F.D. 072_03*	(W 2018-0002210)
Potentilla puberula Krašan	073	Italy	Veneto	*Alonso-Marcos, H., Dobeš, C.*	*Nardi, F.D.*	*Nardi, F.D. 073_06*	(W 2018-0002050)
Potentilla puberula Krašan	074	Italy	Trentino-Alto Adige	*Tribsch, A., Nardi, F.D.*	*Nardi, F.D.*	*Nardi, F.D. 074_02*	(W 2018-0002211)
Potentilla puberula Krašan	074	Italy	Trentino-Alto Adige	*Tribsch, A., Nardi, F.D.*	*Nardi, F.D.*	*Nardi, F.D. 074_03*	(W 2018-0002208)
Potentilla puberula Krašan	075	Italy	Trentino-Alto Adige	*Alonso-Marcos, H., Nardi, F.D., Dobeš, C.*	*Nardi, F.D.*	*Nardi, F.D. 075_13*	(W 2018-0002069)
Potentilla puberula Krašan	076	Italy	Trentino-Alto Adige	*Tribsch, A.*	*Nardi, F.D.*	*Nardi, F.D. 076_08*	(W 2018-0002209)
Potentilla puberula Krašan	078	Italy	Trentino-Alto Adige	*Tribsch, A., Nardi, F.D.*	*Nardi, F.D.*	*Nardi, F.D. 078_08*	(W 2018-0002206)
Potentilla puberula Krašan	078	Italy	Trentino-Alto Adige	*Tribsch, A., Nardi, F.D.*	*Nardi, F.D.*	*Nardi, F.D. 078_10*	(W 2018-0002207)
Potentilla puberula Krašan	079	Italy	Trentino-Alto Adige	*Alonso-Marcos, H., Dobeš, C.*	*Nardi, F.D.*	*Nardi, F.D. 079_02*	(W 2018-0002049)
Potentilla puberula Krašan	080	Italy	Trentino-Alto Adige	*Alonso-Marcos, H., Dobeš, C.*	*Nardi, F.D.*	*Nardi, F.D. 080_02*	(W 2018-0002205)
Potentilla puberula Krašan	080	Italy	Trentino-Alto Adige	*Alonso-Marcos, H., Dobeš, C.*	*Nardi, F.D.*	*Nardi, F.D. 080_03*	(W 2018-0002204)
Potentilla puberula Krašan	080	Italy	Trentino-Alto Adige	*Alonso-Marcos, H., Dobeš, C.*	*Nardi, F.D.*	*Nardi, F.D. 080_06*	(W 2018-0002070)
Potentilla puberula Krašan	080	Italy	Trentino-Alto Adige	*Alonso-Marcos, H., Dobeš, C.*	*Nardi, F.D.*	*Nardi, F.D. 080_12*	(W 2018-0002202)
Potentilla puberula Krašan	082	Italy	Trentino-Alto Adige	*Alonso-Marcos, H., Nardi, F.D.*	*Nardi, F.D.*	*Nardi, F.D. 082_17*	(W 2018-0002203)
Potentilla puberula Krašan	083	Italy	Trentino-Alto Adige	*Alonso-Marcos, H., Nardi, F.D.*	*Nardi, F.D.*	*Nardi, F.D. 083_02*	(W 2018-0002201)
Potentilla puberula Krašan	083	Italy	Trentino-Alto Adige	*Alonso-Marcos, H., Nardi, F.D.*	*Nardi, F.D.*	*Nardi, F.D. 083_20*	(W 2018-0002200)
Potentilla puberula Krašan	084	Italy	Trentino-Alto Adige	*Tribsch, A., Nardi, F.D.*	*Nardi, F.D.*	*Nardi, F.D. 084_01*	(W 2018-0002199)
Potentilla puberula Krašan	084	Italy	Trentino-Alto Adige	*Tribsch, A., Nardi, F.D.*	*Nardi, F.D.*	*Nardi, F.D. 084_12*	(W 2018-0002198)
Potentilla puberula Krašan	084	Italy	Trentino-Alto Adige	*Tribsch, A., Nardi, F.D.*	*Nardi, F.D.*	*Nardi, F.D. 084_16*	(W 2018-0002197)
Potentilla puberula Krašan	085	Italy	Trentino-Alto Adige	*Tribsch, A., Nardi, F.D.*	*Nardi, F.D.*	*Nardi, F.D. 085_03*	(W 2018-0002196)
Potentilla puberula Krašan	085	Italy	Trentino-Alto Adige	*Tribsch, A., Nardi, F.D.*	*Nardi, F.D.*	*Nardi, F.D. 085_08*	(W 2018-0002195)

Table S1. Herbarium vouchers representing studied taxa and deposited in W and GOET.

Taxon	Population	Country	Province	Original collectors	Collector ex cult.	Collection number	Herbarium
Potentilla puberula Krašan	085	Italy	Trentino-Alto Adige	*Tribsch, A., Nardi, F.D.*	*Nardi, F.D.*	*Nardi, F.D. 085_12*	(W 2018-0002146)
Potentilla puberula Krašan	086	Italy	Trentino-Alto Adige	*Alonso-Marcos, H., Nardi, F.D.*	*Nardi, F.D.*	*Nardi, F.D. 086_19*	(W 2018-0002194)
Potentilla puberula Krašan	086	Italy	Trentino-Alto Adige	*Alonso-Marcos, H., Nardi, F.D.*	*Nardi, F.D.*	*Nardi, F.D. 086_20*	(W 2018-0002193)
Potentilla puberula Krašan	086	Italy	Trentino-Alto Adige	*Alonso-Marcos, H., Nardi, F.D.*	*Nardi, F.D.*	*Nardi, F.D. 086_22*	(W 2018-0002192)
Potentilla puberula Krašan	087	Italy	Trentino-Alto Adige	*Alonso-Marcos, H., Nardi, F.D.*	*Nardi, F.D.*	*Nardi, F.D. 087_01*	(W 2018-0002191)
Potentilla puberula Krašan	087	Italy	Trentino-Alto Adige	*Alonso-Marcos, H., Nardi, F.D.*	*Nardi, F.D.*	*Nardi, F.D. 087_20*	(W 2018-0002189)
Potentilla puberula Krašan	088	Italy	Trentino-Alto Adige	*Alonso-Marcos, H., Nardi, F.D.*	*Nardi, F.D.*	*Nardi, F.D. 088_08*	(W 2018-0002190)
Potentilla puberula Krašan	088	Italy	Trentino-Alto Adige	*Alonso-Marcos, H., Nardi, F.D.*	*Nardi, F.D.*	*Nardi, F.D. 088_14*	(W 2018-0002188)
Potentilla puberula Krašan	089	Italy	Trentino-Alto Adige	*Alonso-Marcos, H., Nardi, F.D.*	*Nardi, F.D.*	*Nardi, F.D. 089_01*	(W 2018-0002186)
Potentilla puberula Krašan	090	Italy	Trentino-Alto Adige	*Alonso-Marcos, H., Nardi, F.D.*	*Nardi, F.D.*	*Nardi, F.D. 090_02*	(W 2018-0002187)
Potentilla puberula Krašan	090	Italy	Trentino-Alto Adige	*Alonso-Marcos, H., Nardi, F.D.*	*Nardi, F.D.*	*Nardi, F.D. 090_06*	(W 2018-0002185)
Potentilla puberula Krašan	091	Italy	Trentino-Alto Adige	*Alonso-Marcos, H., Nardi, F.D.*	*Nardi, F.D.*	*Nardi, F.D. 091_07*	(W 2018-0002184)
Potentilla puberula Krašan	092	Italy	Trentino-Alto Adige	*Alonso-Marcos, H., Nardi, F.D.*	*Nardi, F.D.*	*Nardi, F.D. 092_05*	(W 2018-0002182)
Potentilla puberula Krašan	092	Italy	Trentino-Alto Adige	*Alonso-Marcos, H., Nardi, F.D.*	*Nardi, F.D.*	*Nardi, F.D. 092_10*	(W 2018-0002181)
Potentilla puberula Krašan	093	Italy	Trentino-Alto Adige	*Alonso-Marcos, H., Nardi, F.D.*	*Nardi, F.D.*	*Nardi, F.D. 093_02*	(W 2018-0002183)
Potentilla puberula Krašan	094	Italy	Trentino-Alto Adige	*Alonso-Marcos, H., Nardi, F.D.*	*Nardi, F.D.*	*Nardi, F.D. 094_03*	(W 2018-0002180)
Potentilla puberula Krašan	095	Italy	Trentino-Alto Adige	*Alonso-Marcos, H., Nardi, F.D.*	*Nardi, F.D.*	*Nardi, F.D. 095_10*	(W 2018-0002179)
Potentilla puberula Krašan	096	Italy	Trentino-Alto Adige	*Alonso-Marcos, H., Nardi, F.D.*	*Nardi, F.D.*	*Nardi, F.D. 096_02*	(W 2018-0002177)
Potentilla puberula Krašan	096	Italy	Trentino-Alto Adige	*Alonso-Marcos, H., Nardi, F.D.*	*Nardi, F.D.*	*Nardi, F.D. 096_10*	(W 2018-0002147)
Potentilla puberula Krašan	097	Italy	Trentino-Alto Adige	*Alonso-Marcos, H., Nardi, F.D.*	*Nardi, F.D.*	*Nardi, F.D. 097_03*	(W 2018-0002178)
Potentilla puberula Krašan	098	Italy	Trentino-Alto Adige	*Alonso-Marcos, H., Nardi, F.D.*	*Nardi, F.D.*	*Nardi, F.D. 098_02*	(W 2018-0002175)
Potentilla puberula Krašan	098	Italy	Trentino-Alto Adige	*Alonso-Marcos, H., Nardi, F.D.*	*Nardi, F.D.*	*Nardi, F.D. 098_07*	(W 2018-0002176)
Potentilla puberula Krašan	099	Italy	Trentino-Alto Adige	*Alonso-Marcos, H., Nardi, F.D.*	*Nardi, F.D.*	*Nardi, F.D. 099_09*	(W 2018-0002173)
Potentilla puberula Krašan	099	Italy	Trentino-Alto Adige	*Alonso-Marcos, H., Nardi, F.D.*	*Nardi, F.D.*	*Nardi, F.D. 099_18*	(W 2018-0002174)
Potentilla puberula Krašan	100	Italy	Trentino-Alto Adige	*Alonso-Marcos, H., Nardi, F.D.*	*Nardi, F.D.*	*Nardi, F.D. 100_01*	(W 2018-0002170)

Table S1. Herbarium vouchers representing studied taxa and deposited in W and GOET.

Taxon	Population	Country	Province	Original collectors	Collector ex cult.	Collection number	Herbarium
Potentilla puberula Krašan	100	Italy	Trentino-Alto Adige	*Alonso-Marcos, H., Nardi, F.D.*	*Nardi, F.D.*	*Nardi, F.D. 100_02*	(W 2018-0002172)
Potentilla puberula Krašan	100	Italy	Trentino-Alto Adige	*Alonso-Marcos, H., Nardi, F.D.*	*Nardi, F.D.*	*Nardi, F.D. 100_17*	(W 2018-0002171)
Potentilla puberula Krašan	101	Italy	Trentino-Alto Adige	*Alonso-Marcos, H., Nardi, F.D.*	*Nardi, F.D.*	*Nardi, F.D. 101_09*	(W 2018-0002169)
Potentilla puberula Krašan	102	Italy	Trentino-Alto Adige	*Alonso-Marcos, H., Nardi, F.D.*	*Nardi, F.D.*	*Nardi, F.D. 102_06*	(W 2018-0002168)
Potentilla puberula Krašan	103	Italy	Trentino-Alto Adige	*Alonso-Marcos, H., Nardi, F.D.*	*Nardi, F.D.*	*Nardi, F.D. 103_06*	(W 2018-0002166)
Potentilla puberula Krašan	103	Italy	Trentino-Alto Adige	*Alonso-Marcos, H., Nardi, F.D.*	*Nardi, F.D.*	*Nardi, F.D. 103_07*	(W 2018-0002165)
Potentilla puberula Krašan	103	Italy	Trentino-Alto Adige	*Alonso-Marcos, H., Nardi, F.D.*	*Nardi, F.D.*	*Nardi, F.D. 103_10*	(W 2018-0002068)
Potentilla puberula Krašan	104	Italy	Trentino-Alto Adige	*Alonso-Marcos, H., Nardi, F.D.*	*Nardi, F.D.*	*Nardi, F.D. 104_11*	(W 2018-0002066)
Potentilla puberula Krašan	104	Italy	Trentino-Alto Adige	*Alonso-Marcos, H., Nardi, F.D.*	*Nardi, F.D.*	*Nardi, F.D. 104_13*	(W 2018-0002046)
Potentilla puberula Krašan	105	Switzerland	Grisons	*Alonso-Marcos, H., Nardi, F.D.*	*Nardi, F.D.*	*Nardi, F.D. 105_08*	(W 2018-0002047)
Potentilla puberula Krašan	109	Italy	Trentino-Alto Adige	*Dobeš, C.*	*Nardi, F.D.*	*Nardi, F.D. 109_03*	(W 2018-0002164)
Potentilla puberula Krašan	110	Italy	Trentino-Alto Adige	*Dobeš, C.*	*Nardi, F.D.*	*Nardi, F.D. 110_01*	(W 2018-0002163)
Potentilla puberula Krašan	111	Italy	Trentino-Alto Adige	*Dobeš, C.*	*Nardi, F.D.*	*Nardi, F.D. 111_01*	(W 2018-0002067)
Potentilla puberula Krašan	111	Italy	Trentino-Alto Adige	*Dobeš, C.*	*Nardi, F.D.*	*Nardi, F.D. 111_04*	(W 2018-0002161)
Potentilla puberula Krašan	111	Italy	Trentino-Alto Adige	*Dobeš, C.*	*Nardi, F.D.*	*Nardi, F.D. 111_06*	(W 2018-0002162)
Potentilla puberula Krašan	111	Italy	Trentino-Alto Adige	*Dobeš, C.*	*Nardi, F.D.*	*Nardi, F.D. 111_15*	(W 2018-0002160)
Potentilla puberula Krašan	112	Italy	Trentino-Alto Adige	*Dobeš, C.*	*Nardi, F.D.*	*Nardi, F.D. 112_06*	(W 2018-0002158)
Potentilla puberula Krašan	113	Italy	Trentino-Alto Adige	*Dobeš, C.*	*Nardi, F.D.*	*Nardi, F.D. 113_04*	(W 2018-0002159)
Potentilla puberula Krašan	115	Italy	Trentino-Alto Adige	*Dobeš, C.*	*Nardi, F.D.*	*Nardi, F.D. 115_01*	(W 2018-0002157)
Potentilla puberula Krašan	115	Italy	Trentino-Alto Adige	*Dobeš, C.*	*Nardi, F.D.*	*Nardi, F.D. 115_03*	(W 2018-0002156)
Potentilla puberula Krašan	115	Italy	Trentino-Alto Adige	*Dobeš, C.*	*Nardi, F.D.*	*Nardi, F.D. 115_04*	(W 2018-0002155)
Potentilla puberula Krašan	116	Italy	Trentino-Alto Adige	*Dobeš, C.*	*Nardi, F.D.*	*Nardi, F.D. 116_03*	(W 2018-0002154)
Potentilla puberula Krašan	117	Italy	Trentino-Alto Adige	*Dobeš, C.*	*Nardi, F.D.*	*Nardi, F.D. 117_03*	(W 2018-0002151)
Potentilla puberula Krašan	117	Italy	Trentino-Alto Adige	*Dobeš, C.*	*Nardi, F.D.*	*Nardi, F.D. 117_04*	(W 2018-0002153)
Potentilla puberula Krašan	117	Italy	Trentino-Alto Adige	*Dobeš, C.*	*Nardi, F.D.*	*Nardi, F.D. 117_07*	(W 2018-0002152)

Table S1. Herbarium vouchers representing studied taxa and deposited in W and GOET.

Taxon	Population	Country	Province	Original collectors	Collector ex cult.	Collection number	Herbarium
Potentilla puberula Krašan	118	Italy	Trentino-Alto Adige	*Dobeš, C.*	*Nardi, F.D.*	*Nardi, F.D. 118_01*	(W 2018-0002150)
Potentilla puberula Krašan	118	Italy	Trentino-Alto Adige	*Dobeš, C.*	*Nardi, F.D.*	*Nardi, F.D. 118_02*	(W 2018-0002149)
Potentilla puberula Krašan	119	Italy	Trentino-Alto Adige	*Dobeš, C.*	*Nardi, F.D.*	*Nardi, F.D. 119_15*	(W 2018-0002111)
Potentilla puberula Krašan	120	Italy	Trentino-Alto Adige	*Dobeš, C.*	*Nardi, F.D.*	*Nardi, F.D. 120_14*	(W 2018-0002145)
Potentilla puberula Krašan	121	Italy	Trentino-Alto Adige	*Dobeš, C.*	*Nardi, F.D.*	*Nardi, F.D. 121_01*	(W 2018-0002065)
Potentilla puberula Krašan	121	Italy	Trentino-Alto Adige	*Dobeš, C.*	*Nardi, F.D.*	*Nardi, F.D. 121_04*	(W 2018-0002144)
Potentilla puberula Krašan	122	Italy	Trentino-Alto Adige	*Dobeš, C.*	*Nardi, F.D.*	*Nardi, F.D. 122_05*	(W 2018-0002062)
Potentilla puberula Krašan	122	Italy	Trentino-Alto Adige	*Dobeš, C.*	*Nardi, F.D.*	*Nardi, F.D. 122_08*	(W 2018-0002107)
Potentilla puberula Krašan	122	Italy	Trentino-Alto Adige	*Dobeš, C.*	*Nardi, F.D.*	*Nardi, F.D. 122_10*	(W 2018-0002143)
Potentilla puberula Krašan	122	Italy	Trentino-Alto Adige	*Dobeš, C.*	*Nardi, F.D.*	*Nardi, F.D. 122_12*	(W 2018-0002142)
Potentilla puberula Krašan	123	Italy	Trentino-Alto Adige	*Dobeš, C.*	*Nardi, F.D.*	*Nardi, F.D. 123_08*	(W 2018-0002141)
Potentilla puberula Krašan	123	Italy	Trentino-Alto Adige	*Dobeš, C.*	*Nardi, F.D.*	*Nardi, F.D. 123_10*	(W 2018-0002140)
Potentilla puberula Krašan	125	Austria	Tyrol	*Alonso-Marcos, H., Nardi, F.D.*	*Nardi, F.D.*	*Nardi, F.D. 125_11*	(W 2018-0002045)
Potentilla puberula Krašan	126	Austria	Tyrol	*Alonso-Marcos, H., Nardi, F.D.*	*Nardi, F.D.*	*Nardi, F.D. 126_09*	(W 2018-0002139)
Potentilla puberula Krašan	127	Austria	Tyrol	*Alonso-Marcos, H., Nardi, F.D.*	*Nardi, F.D.*	*Nardi, F.D. 127_07*	(W 2018-0002138)
Potentilla puberula Krašan	128	Austria	Tyrol	*Alonso-Marcos, H., Nardi, F.D.*	*Nardi, F.D.*	*Nardi, F.D. 128_06*	(W 2018-0002137)
Potentilla puberula Krašan	129	Austria	Tyrol	*Alonso-Marcos, H., Nardi, F.D.*	*Nardi, F.D.*	*Nardi, F.D. 129_11*	(W 2018-0002136)
Potentilla puberula Krašan	130	Austria	Tyrol	*Alonso-Marcos, H., Nardi, F.D.*	*Nardi, F.D.*	*Nardi, F.D. 130_09*	(W 2018-0002134)
Potentilla puberula Krašan	131	Austria	Tyrol	*Alonso-Marcos, H., Nardi, F.D.*	*Nardi, F.D.*	*Nardi, F.D. 131_01*	(W 2018-0002135)
Potentilla puberula Krašan	132	Austria	Tyrol	*Alonso-Marcos, H., Nardi, F.D.*	*Nardi, F.D.*	*Nardi, F.D. 132_02*	(W 2018-0002132)
Potentilla puberula Krašan	133	Austria	Tyrol	*Alonso-Marcos, H., Nardi, F.D.*	*Nardi, F.D.*	*Nardi, F.D. 133_09*	(W 2018-0002133)
Potentilla puberula Krašan	134	Austria	Tyrol	*Alonso-Marcos, H., Nardi, F.D.*	*Nardi, F.D.*	*Nardi, F.D. 134_13*	(W 2018-0002130)
Potentilla puberula Krašan	134	Austria	Tyrol	*Alonso-Marcos, H., Nardi, F.D.*	*Nardi, F.D.*	*Nardi, F.D. 134_15*	(W 2018-0002131)
Potentilla puberula Krašan	135	Austria	Tyrol	*Alonso-Marcos, H., Nardi, F.D.*	*Nardi, F.D.*	*Nardi, F.D. 135_19*	(W 2018-0002128)
Potentilla puberula Krašan	136	Austria	Tyrol	*Alonso-Marcos, H., Nardi, F.D.*	*Nardi, F.D.*	*Nardi, F.D. 136_06*	(W 2018-0002064)

Table S1. Herbarium vouchers representing studied taxa and deposited in W and GOET.

Taxon	Population	Country	Province	Original collectors	Collector ex cult.	Collection number	Herbarium
Potentilla puberula Krašan	137	Austria	Tyrol	*Alonso-Marcos, H., Nardi, F.D.*	*Nardi, F.D.*	*Nardi, F.D. 137_02*	(W 2018-0002129)
Potentilla puberula Krašan	138	Austria	Tyrol	*Alonso-Marcos, H., Nardi, F.D.*	*Nardi, F.D.*	*Nardi, F.D. 138_10*	(W 2018-0002126)
Potentilla puberula Krašan	138	Austria	Tyrol	*Alonso-Marcos, H., Nardi, F.D.*	*Nardi, F.D.*	*Nardi, F.D. 138_11*	(W 2018-0002127)
Potentilla puberula Krašan	139	Austria	Tyrol	*Alonso-Marcos, H., Nardi, F.D.*	*Nardi, F.D.*	*Nardi, F.D. 139_03*	(W 2018-0002124)
Potentilla puberula Krašan	140	Austria	Tyrol	*Alonso-Marcos, H., Nardi, F.D.*	*Nardi, F.D.*	*Nardi, F.D. 140_12*	(W 2018-0002122)
Potentilla puberula Krašan	141	Austria	Tyrol	*Alonso-Marcos, H., Nardi, F.D.*	*Nardi, F.D.*	*Nardi, F.D. 141_01*	(W 2018-0002123)
Potentilla puberula Krašan	142	Austria	Tyrol	*Alonso-Marcos, H., Nardi, F.D.*	*Nardi, F.D.*	*Nardi, F.D. 142_06*	(W 2018-0002121)
Potentilla puberula Krašan	143	Austria	Tyrol	*Alonso-Marcos, H., Nardi, F.D., Stifter, S., Julian Ananda Hainer*	*Nardi, F.D.*	*Nardi, F.D. 143_05*	(W 2018-0002110)
Potentilla puberula Krašan	143	Austria	Tyrol	*Alonso-Marcos, H., Nardi, F.D., Stifter, S., Julian Ananda Hainer*	*Nardi, F.D.*	*Nardi, F.D. 143_06*	(W 2018-0002061)
Potentilla puberula Krašan	145	Italy	Trentino-Alto Adige	*Stifter, S., Haider, J.A.*	*Nardi, F.D.*	*Nardi, F.D. 145_01*	(W 2018-0002119)
Potentilla puberula Krašan	146	Italy	Trentino-Alto Adige	*Stifter, S., Haider, J.A.*	*Nardi, F.D.*	*Nardi, F.D. 146_03*	(W 2018-0002118)
Potentilla puberula Krašan	146	Italy	Trentino-Alto Adige	*Stifter, S., Haider, J.A.*	*Nardi, F.D.*	*Nardi, F.D. 146_07*	(W 2018-0002063)
Potentilla puberula Krašan	146	Italy	Trentino-Alto Adige	*Stifter, S., Haider, J.A.*	*Nardi, F.D.*	*Nardi, F.D. 146_20*	(W 2018-0002120)
Potentilla puberula Krašan	148	Italy	Trentino-Alto Adige	*Stifter, S., Haider, J.A.*	*Nardi, F.D.*	*Nardi, F.D. 148_05*	(W 2018-0002117)
Potentilla puberula Krašan	149	Italy	Trentino-Alto Adige	*Stifter, S., Haider, J.A.*	*Nardi, F.D.*	*Nardi, F.D. 149_13*	(W 2018-0002060)
Potentilla puberula Krašan	149	Italy	Trentino-Alto Adige	*Stifter, S., Haider, J.A.*	*Nardi, F.D.*	*Nardi, F.D. 149_14*	(W 2018-0002058)
Potentilla puberula Krašan	150	Italy	Trentino-Alto Adige	*Stifter, S., Haider, J.A.*	*Nardi, F.D.*	*Nardi, F.D. 150_05*	(W 2018-0002115)
Potentilla puberula Krašan	150	Italy	Trentino-Alto Adige	*Stifter, S., Haider, J.A.*	*Nardi, F.D.*	*Nardi, F.D. 150_13*	(W 2018-0002114)
Potentilla puberula Krašan	150	Italy	Trentino-Alto Adige	*Stifter, S., Haider, J.A.*	*Nardi, F.D.*	*Nardi, F.D. 150_18*	(W 2018-0002113)
Potentilla puberula Krašan	151	Italy	Trentino-Alto Adige	*Stifter, S., Haider, J.A.*	*Nardi, F.D.*	*Nardi, F.D. 151_03*	(W 2018-0002059)
Potentilla puberula Krašan	152	Italy	Trentino-Alto Adige	*Stifter, S., Haider, J.A.*	*Nardi, F.D.*	*Nardi, F.D. 152_03*	(W 2018-0002044)
Potentilla puberula Krašan	153	Italy	Trentino-Alto Adige	*Stifter, S., Haider, J.A.*	*Nardi, F.D.*	*Nardi, F.D. 153_08*	(W 2018-0002108)
Potentilla puberula Krašan	154	Italy	Trentino-Alto Adige	*Stifter, S., Haider, J.A.*	*Nardi, F.D.*	*Nardi, F.D. 154_07*	(W 2018-0002105)
Potentilla puberula Krašan	156	Austria	Tyrol	*Alonso-Marcos, H., Nardi, F.D.*	*Nardi, F.D.*	*Nardi, F.D. 156_01*	(W 2018-0002104)

Table S1. Herbarium vouchers representing studied taxa and deposited in W and GOET.

Taxon	Population	Country	Province	Original collectors	Collector ex cult.	Collection number	Herbarium
Potentilla puberula Krašan	156	Austria	Tyrol	*Alonso-Marcos, H., Nardi, F.D.*	*Nardi, F.D.*	*Nardi, F.D. 156_02*	(W 2018-0002103)
Potentilla puberula Krašan	157	Austria	Tyrol	*Alonso-Marcos, H., Nardi, F.D.*	*Nardi, F.D.*	*Nardi, F.D. 157_02*	(W 2018-0002106)
Potentilla puberula Krašan	157	Austria	Tyrol	*Alonso-Marcos, H., Nardi, F.D.*	*Nardi, F.D.*	*Nardi, F.D. 157_12*	(W 2018-0002101)
Potentilla puberula Krašan	158	Austria	Tyrol	*Alonso-Marcos, H., Nardi, F.D.*	*Nardi, F.D.*	*Nardi, F.D. 158_07*	(W 2018-0002102)
Potentilla puberula Krašan	159	Austria	Tyrol	*Alonso-Marcos, H., Nardi, F.D.*	*Nardi, F.D.*	*Nardi, F.D. 159_04*	(W 2018-0002100)
Potentilla puberula Krašan	159	Austria	Tyrol	*Alonso-Marcos, H., Nardi, F.D.*	*Nardi, F.D.*	*Nardi, F.D. 159_16*	(W 2018-0002099)
Potentilla puberula Krašan	159	Austria	Tyrol	*Alonso-Marcos, H., Nardi, F.D.*	*Nardi, F.D.*	*Nardi, F.D. 159_18*	(W 2018-0002096)
Potentilla puberula Krašan	160	Austria	Tyrol	*Alonso-Marcos, H., Nardi, F.D.*	*Nardi, F.D.*	*Nardi, F.D. 160_07*	(W 2018-0002097)
Potentilla puberula Krašan	160	Austria	Tyrol	*Alonso-Marcos, H., Nardi, F.D.*	*Nardi, F.D.*	*Nardi, F.D. 160_13*	(W 2018-0002094)
Potentilla puberula Krašan	161	Austria	Tyrol	*Alonso-Marcos, H., Nardi, F.D.*	*Nardi, F.D.*	*Nardi, F.D. 161_02*	(W 2018-0002042)
Potentilla puberula Krašan	162	Austria	Tyrol	*Alonso-Marcos, H., Nardi, F.D.*	*Nardi, F.D.*	*Nardi, F.D. 162_07*	(W 2018-0002053)
Potentilla puberula Krašan	163	Austria	Tyrol	*Alonso-Marcos, H., Nardi, F.D.*	*Nardi, F.D.*	*Nardi, F.D. 163_01*	(W 2018-0002095)
Potentilla puberula Krašan	164	Italy	Trentino-Alto Adige	*Stifter, S., Haider, J.A.*	*Nardi, F.D.*	*Nardi, F.D. 164_01*	(W 2018-0002098)
Potentilla puberula Krašan	164	Italy	Trentino-Alto Adige	*Stifter, S., Haider, J.A.*	*Nardi, F.D.*	*Nardi, F.D. 164_04*	(W 2018-0002057)
Potentilla puberula Krašan	165	Italy	Trentino-Alto Adige	*Stifter, S., Haider, J.A.*	*Nardi, F.D.*	*Nardi, F.D. 165_03*	(W 2018-0002093)
Potentilla puberula Krašan	165	Italy	Trentino-Alto Adige	*Stifter, S., Haider, J.A.*	*Nardi, F.D.*	*Nardi, F.D. 165_11*	(W 2018-0002091)
Potentilla puberula Krašan	165	Italy	Trentino-Alto Adige	*Stifter, S., Haider, J.A.*	*Nardi, F.D.*	*Nardi, F.D. 165_18*	(W 2018-0002092)
Potentilla puberula Krašan	169	Italy	Veneto	*Dobeš, C.*	*Nardi, F.D.*	*Nardi, F.D. 169_02*	(W 2018-0002056)
Potentilla puberula Krašan	169	Italy	Veneto	*Dobeš, C.*	*Nardi, F.D.*	*Nardi, F.D. 169_15*	(W 2018-0002090)
Potentilla puberula Krašan	170	Italy	Veneto	*Dobeš, C.*	*Nardi, F.D.*	*Nardi, F.D. 170_02*	(W 2018-0002089)
Potentilla puberula Krašan	170	Italy	Veneto	*Dobeš, C.*	*Nardi, F.D.*	*Nardi, F.D. 170_05*	(W 2018-0002055)
Potentilla puberula Krašan	170	Italy	Veneto	*Dobeš, C.*	*Nardi, F.D.*	*Nardi, F.D. 170_14*	(W 2018-0002054)
Potentilla puberula Krašan	171	Italy	Veneto	*Dobeš, C.*	*Nardi, F.D.*	*Nardi, F.D. 171_01*	(W 2018-0002088)
Potentilla puberula Krašan	172	Italy	Veneto	*Dobeš, C.*	*Nardi, F.D.*	*Nardi, F.D. 172_04*	(W 2018-0002043)
Potentilla puberula Krašan	172	Italy	Veneto	*Dobeš, C.*	*Nardi, F.D.*	*Nardi, F.D. 172_13*	(W 2018-0002041)

Table S1. Herbarium vouchers representing studied taxa and deposited in W and GOET.

Taxon	Population	Country	Province	Original collectors	Collector ex cult.	Collection number	Herbarium
Potentilla puberula Krašan	172	Italy	Veneto	*Dobeš, C.*	*Nardi, F.D.*	*Nardi, F.D. 172_18*	(W 2018-0002087)
Potentilla puberula Krašan	172	Italy	Veneto	*Dobeš, C.*	*Nardi, F.D.*	*Nardi, F.D. 172_20*	(W 2018-0002040)
Potentilla puberula Krašan	173	Italy	Friuli-Venezia Giulia	*Dobeš, C.*	*Nardi, F.D.*	*Nardi, F.D. 173_03*	(W 2018-0002086)
Potentilla puberula Krašan	173	Italy	Friuli-Venezia Giulia	*Dobeš, C.*	*Nardi, F.D.*	*Nardi, F.D. 173_08*	(W 2018-0002039)
Potentilla puberula Krašan	173	Italy	Friuli-Venezia Giulia	*Dobeš, C.*	*Nardi, F.D.*	*Nardi, F.D. 173_14*	(W 2018-0002037)
Potentilla puberula Krašan	174	Italy	Friuli-Venezia Giulia	*Dobeš, C.*	*Nardi, F.D.*	*Nardi, F.D. 174_01*	(W 2018-0002085)
Potentilla puberula Krašan	175	Italy	Friuli-Venezia Giulia	*Dobeš, C.*	*Nardi, F.D.*	*Nardi, F.D. 175_06*	(W 2018-0002083)
Potentilla puberula Krašan	179	Austria	Carinthia	*Dobeš, C.*	*Nardi, F.D.*	*Nardi, F.D. 179_02*	(W 2018-0002082)
Potentilla puberula Krašan	179	Austria	Carinthia	*Dobeš, C.*	*Nardi, F.D.*	*Nardi, F.D. 179_04*	(W 2018-0002081)
Potentilla puberula Krašan	180	Italy	Friuli-Venezia Giulia	*Dobeš, C.*	*Nardi, F.D.*	*Nardi, F.D. 180_02*	(W 2018-0002078)
Potentilla puberula Krašan	180	Italy	Friuli-Venezia Giulia	*Dobeš, C.*	*Nardi, F.D.*	*Nardi, F.D. 180_06*	(W 2018-0002080)
Potentilla puberula Krašan	181	Italy	Friuli-Venezia Giulia	*Dobeš, C.*	*Nardi, F.D.*	*Nardi, F.D. 181_04*	(W 2018-0002079)
Potentilla puberula Krašan	183	Italy	Friuli-Venezia Giulia	*Dobeš, C.*	*Nardi, F.D.*	*Nardi, F.D. 183_09*	(W 2018-0002052)
Potentilla puberula Krašan	183	Italy	Friuli-Venezia Giulia	*Dobeš, C.*	*Nardi, F.D.*	*Nardi, F.D. 183_22*	(W 2018-0002077)
Potentilla puberula Krašan	184	Italy	Friuli-Venezia Giulia	*Dobeš, C.*	*Nardi, F.D.*	*Nardi, F.D. 184_06*	(W 2018-0002074)
Potentilla puberula Krašan	185	Italy	Friuli-Venezia Giulia	*Dobeš, C.*	*Nardi, F.D.*	*Nardi, F.D. 185_01*	(W 2018-0002073)
Potentilla puberula Krašan	185	Italy	Friuli-Venezia Giulia	*Dobeš, C.*	*Nardi, F.D.*	*Nardi, F.D. 185_05*	(W 2018-0002072)
Potentilla puberula Krašan	186	Austria	Carinthia	*Dobeš, C.*	*Nardi, F.D.*	*Nardi, F.D. 186_03*	(W 2018-0002071)
Potentilla puberula Krašan	186	Austria	Carinthia	*Dobeš, C.*	*Nardi, F.D.*	*Nardi, F.D. 186_05*	(W 2018-0002051)

Table S2. GenBank (Benson & al., 2005) codes of *rps16* and *trnH-psbA* cpDNA regions in *Potentilla puberula* Krašan reported by combined haplotype.

Haplotype code	*rps16*	*trnH-psbA*
H1	MG995594	MG995643
H2	MG995595	MG995644
H3	MG995596	MG995645
H4	MG995597	MG995646
H5	MG995598	MG995647
H6	MG995599	MG995648
H7	MG995600	MG995649
H8	MG995601	MG995650
H9	MG995602	MG995651
H10	MG995603	MG995652
H11	MG995604	MG995653
H12	MG995605	MG995654
H13	MG995606	MG995655
H14	MG995607	MG995656
H15	MG995608	MG995657
H16	MG995609	MG995658
H17	MG995610	MG995659
H18	MG995611	MG995660
H19	MG995612	MG995661
H20	MG995613	MG995662
H21	MG995614	MG995663
H22	MG995615	MG995664
H23	MG995616	MG995665
H24	MG995617	MG995666
H25	MG995618	MG995667
H26	MG995619	MG995668
H27	MG995620	MG995669
H28	MG995621	MG995670
H29	MG995622	MG995671
H30	MG995623	MG995672
H31	MG995624	MG995673
H32	MG995625	MG995674
H33	MG995626	MG995675
H34	MG995627	MG995676
H35	MG995628	MG995677
H36	MG995629	MG995678
H37	MG995630	MG995679
H38	MG995631	MG995680
H39	MG995632	MG995681
H40	MG995633	MG995682
H41	MG995634	MG995683
H42	MG995635	MG995684
H43	MG995636	MG995685
H44	MG995637	MG995686
H45	MG995638	MG995687
H46	MG995639	MG995688
H47	MG995640	MG995689
H48	MG995641	MG995690
H49	MG995642	MG995691

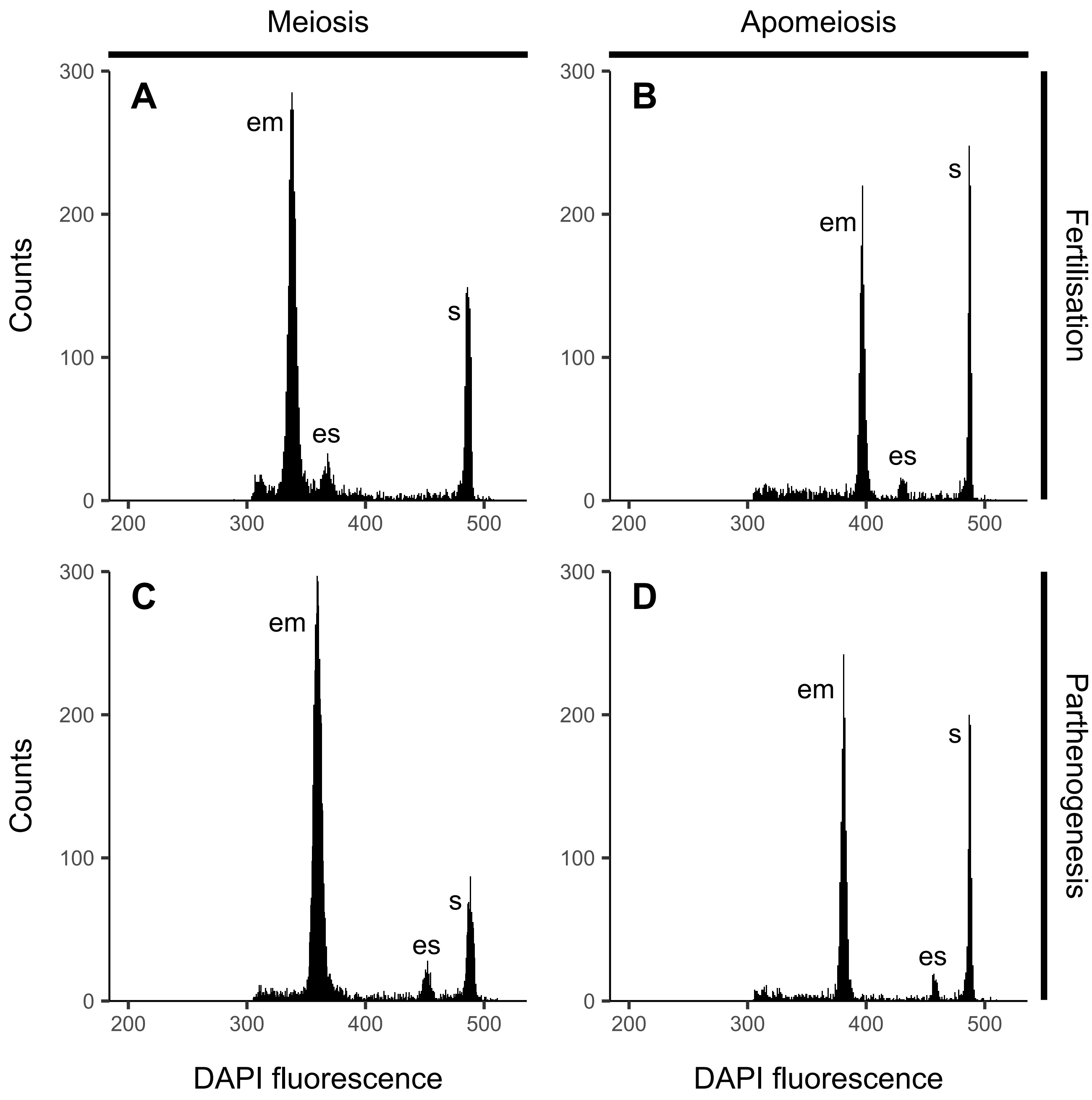

Fig. S1. Examples of reproductive modes of seed formation in *Potentilla puberula* Krašan as observed by flow cytometric seed screen. **A,** Regular sexuality in a tetraploid individual; **B,** Irregular sexuality (B_{III} hybrids formation) in a pentaploid individual; **C,** Haploid parthenogenesis in an octoploid individual; **D,** Apomixis in a heptaploid individual. — em: embryo; es: endosperm; s: standard.

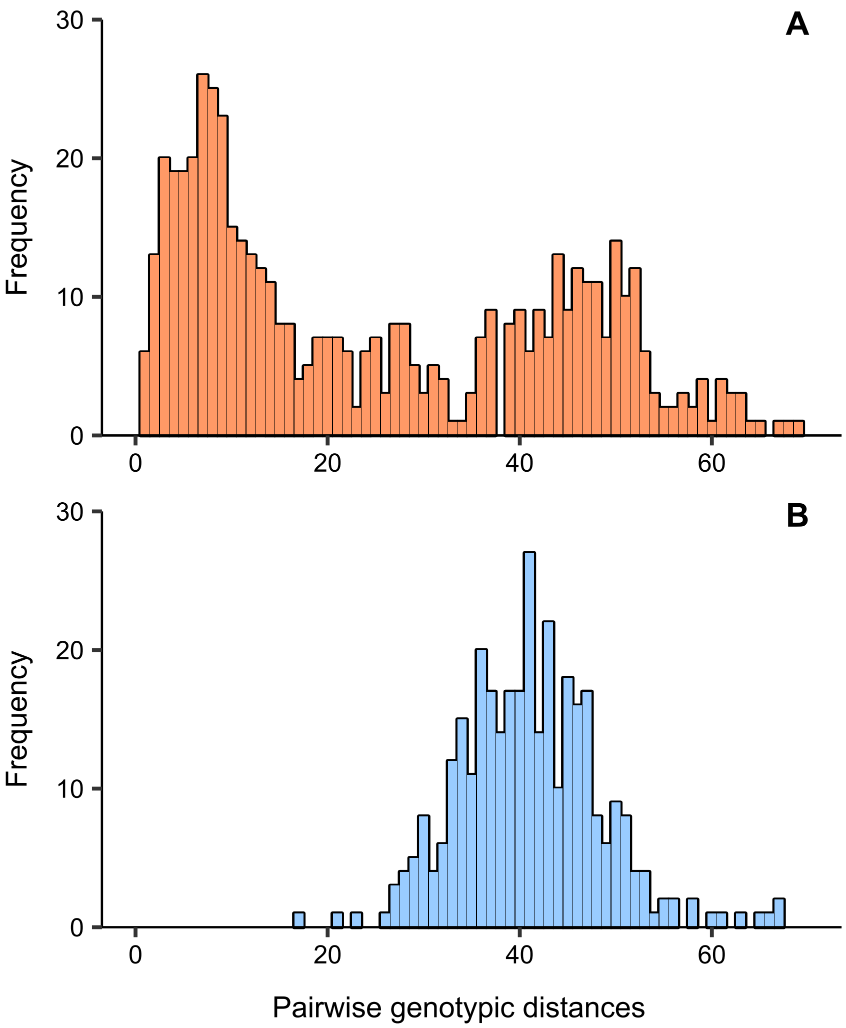

Fig. S2. Distribution of the pairwise genotypic distances (simple matching) among penta- to octoploid **(A)** and tetraploid **(B)** individuals of the same ploidy and same geographic population in *Potentilla puberula* Krašan.

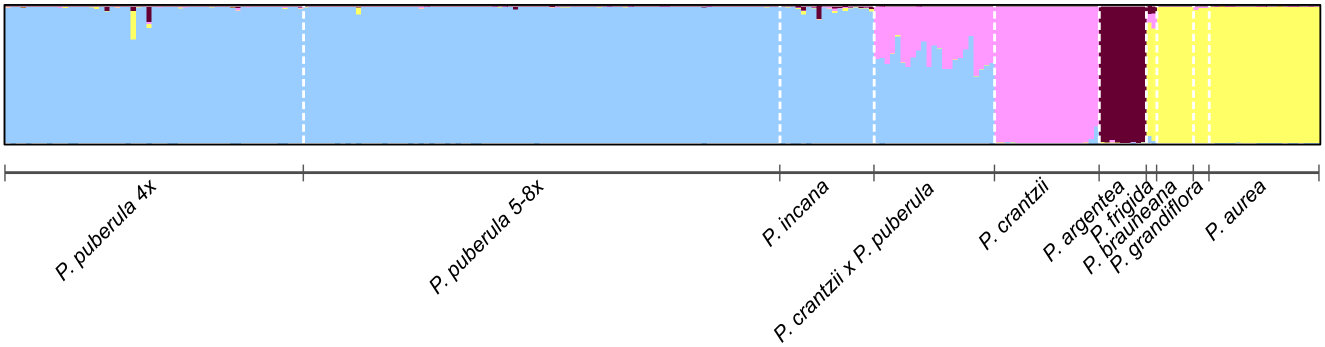

Fig. S3. Individual cluster assignment of 251 single genotypes of *Potentilla* L. taxa resulting from a STRUCTURE analysis based on 335 AFLP markers with K = 4, according to 8/10 runs with lower likelihood.

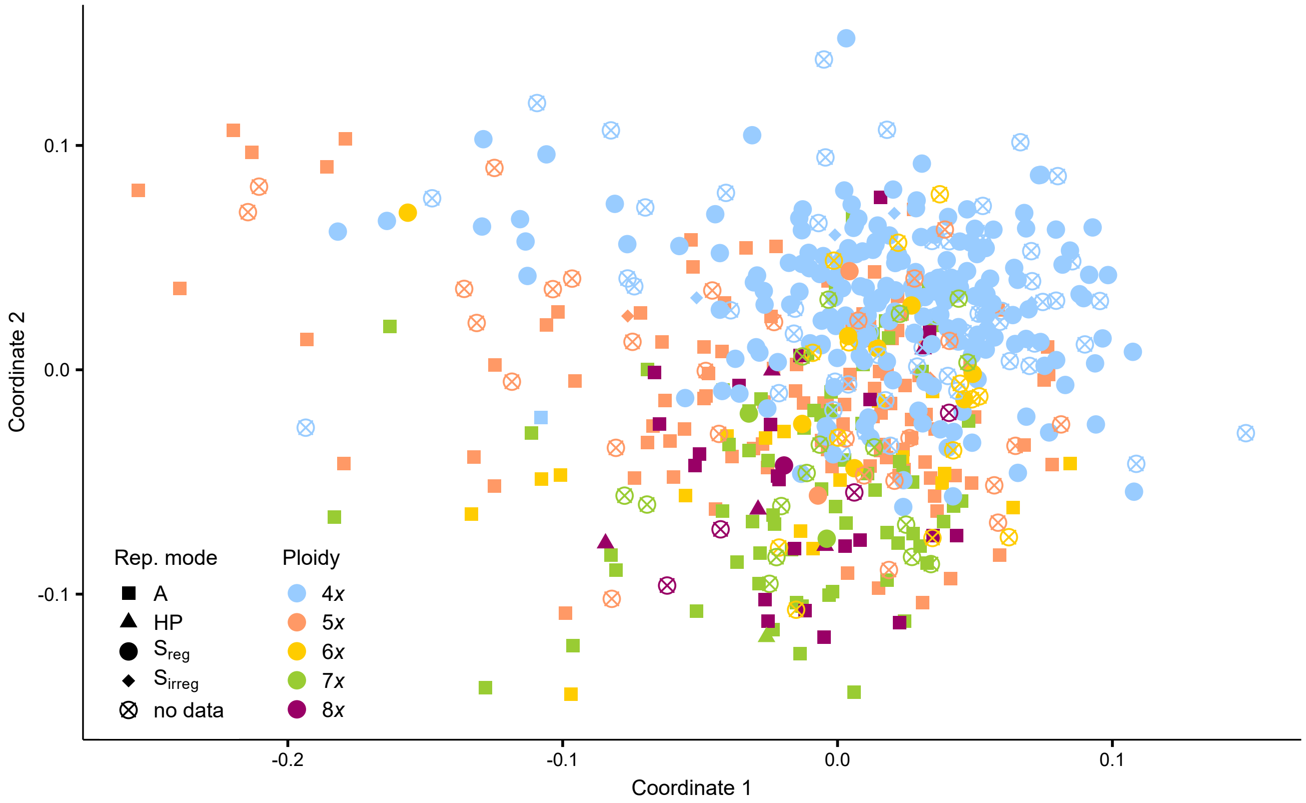

Fig. S4. Principal coordinate analysis of 554 unique genotypes of *Potentilla puberula* Krašan based on 367 AFLP markers, after removal of AFLP fragments 170 VIC, 219 FAM and 286 FAM. The coordinates 1 and 2 explain 2.81% and 2.16% of the total genetic variation, respectively. A: apomixis; HP: haploid parthenogenesis; S_{irreg}: irregular sexuality (B_{III} hybrids formation); S_{reg}: regular sexuality.

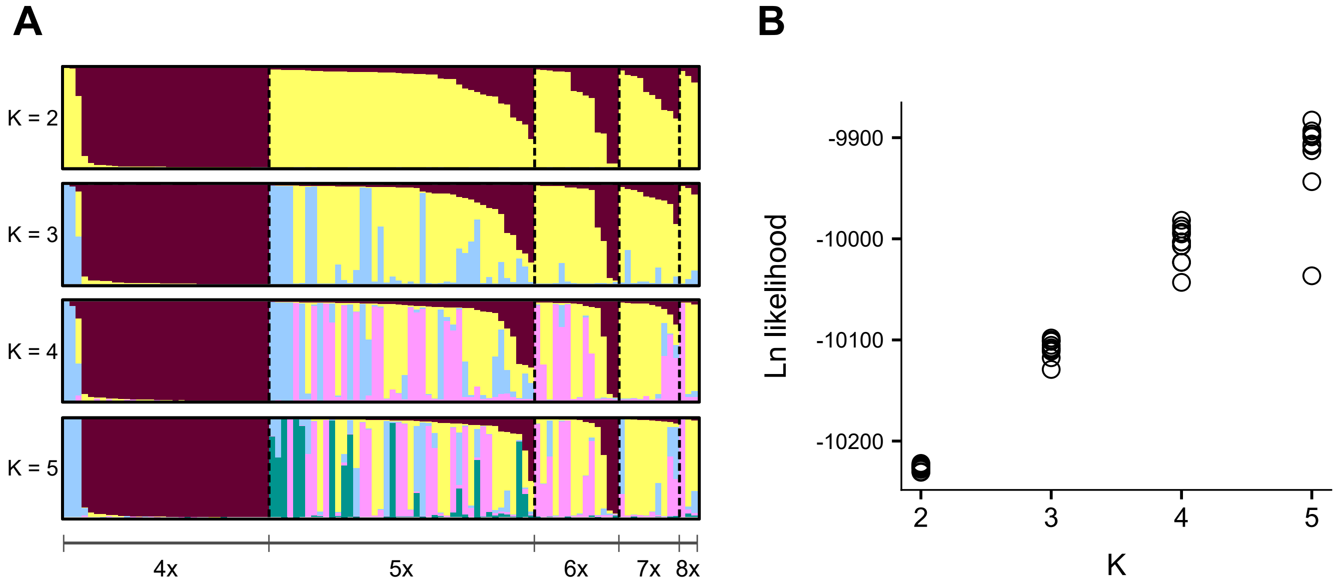

Fig. S5. Results of the STRUCTURE analysis conducted on 105 unique genotypes of *Potentilla puberula* Krašan from Eastern Tyrol populations and based on 252 polymorphic markers. **A,** Individual cluster membership to Ks from 2 to 5; **B,** Logarithmic likelihood of single runs per K value.
