# Supplementary material for: Sexual intraspecific recombination but not de novo origin governs the genesis of new apomictic genotypes in Potentilla puberula (Rosaceae)
Source: Taxon. Author manuscript; Available in PMC 2019 Feb 20. (PMC6382066)
Supplement: Appendix [file NIHMS81441-supplement-Appendix.pdf]

Appendix 1. Studied populations, ploidy levels and reproductive modes of *Potentilla puberula* Krašan and seven closely-related species plus one putative hybrid.

ID	Taxon	Lat. (°N)	Long. (°E)	Population	Country[a]	Elevation (m a.s.l.)	Collectors[b]	Ploidy levels	N	N_g	N_s	A	HP	S_{reg}	S_{irreg}	N_i
5[c]	*P. puberula*	47.00887	12.4664	Rabenstein	A	1380	JAH & SS	5*x*	12	4	2	2				1
6[c]	*P. puberula*	47.00477	12.43528	Obermauern	A	1300	JAH & SS	4*x*	11	4	4			4		
								6*x*	1	1	1			1		
7[c]	*P. puberula*	46.75024	12.43778	Heinfels Schloss	A	1100	JAH & SS	4*x*	11	4	1			1		
								7*x*	5	4						
9[c,d]	*P. puberula*	46.78128	12.5748	Raut	A	1450	JAH & SS	4*x*	5	3	41			41		
								5*x*	4	4	31	29		2		
13[c,d]	*P. puberula*	47.01812	12.33223	Groder	A	1520	JAH & SS	4*x*	5	2	14			13	1	1
								5*x*	4	3	31	30			1	
								6*x*	1	1						1
15[c,d]	*P. puberula*	46.98602	12.63151	Lana S	A	1295	JAH & SS	5*x*	5	3	1	1				
								6*x*	4	3	52	49	1	1	1	
								8*x*	1	1						1
16[c]	*P. puberula*	46.92665	12.4221	St. Veit	A	1480	JAH & SS	5*x*	11	4	5	5				
17[c,d]	*P. puberula*	47.02602	12.5281	Stein	A	1300	JAH & SS	5*x*	3	3	8	7			1	
								7*x*	2	2	10	9		1		
								8*x*	4	4	13	12		1		
22[c]	*P. puberula*	47.05703	12.49057	Katalalm	A	1720	JAH & SS	5*x*	12	4	3	3				
26[c]	*P. puberula*	47.01067	12.3059	Pebellalm	A	1580	JAH & SS	5*x*	11	4	6	5		1		1
29[c]	*P. puberula*	46.98468	12.539	Bichl	A	940	JAH & SS	4*x*	5	4	3			2	1	4
								5*x*	6	4	2	2				2
								7*x*	1	1	1	1				1
31[c]	*P. puberula*	47.02117	12.37452	Prägraten N	A	1400	JAH & SS	4*x*	12	4	4			4		
39[c]	*P. puberula*	46.92542	12.53903	Ratzell	A	1190	JAH & SS	5*x*	12	3	1	1				
40[c]	*P. puberula*	46.78937	12.63775	Oberassling	A	1220	JAH & SS	5*x*	9	4	4	3			1	
								6*x*	4	4	3	3				
								7*x*	6	4	4	4				
43[c]	*P. puberula*	46.92237	12.33858	Erlach Gehöft	A	1610	JAH & SS	5*x*	11	3	4	4				
45[c]	*P. puberula*	47.01787	12.40872	Obersteiner	A	1460	JAH & SS	4*x*	11	4	2			2		2
								5*x*	4	4	4	4				1
								6*x*	1							
								7*x*	2		2	2				
54[c]	*P. puberula*	46.78878	12.58503	St. Justina	A	1200	JAH & SS	4*x*	1	1	1	1				1
								5*x*	8	4	1	1				2
61	*P. puberula*	45.93125	10.89694	Arco NE	I	110	HAM & FDN	4*x*	19	4	2			2		
62	*P. puberula*	45.55506	10.82355	Gaium Croce	I	180	HAM, CD, FDN & AT	4*x*	14	4	4			4		
63	*P. puberula*	45.64632	10.87395	Brenito Belluno	I	230	HAM, CD, FDN & AT	4*x*	16	4	3			3		
64	*P. puberula*	45.67443	10.90949	Ossenigo	I	250	HAM, CD, FDN & AT	4*x*	9	5	4			4		1
								5*x*	4	4	4	4				1
								7*x*	1	1	3	1		2		1
								8*x*	1	1	1		1			1
65	*P. puberula*	45.86729	11.18823	Zoreri	I	900	HAM & CD	5*x*	7	4	4	4				2
								7*x*	4	4	3	3				1
66	*P. puberula*	45.92031	11.13336	Mezzomonte	I	610	HAM & CD	5*x*	9	6	2	2				
								7*x*	9	4	4	4				
								8*x*	1	1	1	1				
67	*P. puberula*	46.20694	11.15578	Monreale	I	460	HAM & CD	4*x*	18	3	1			1		
								6*x*	1	1						
68	*P. puberula*	45.53424	10.36401	Scaiola	I	200	HAM & CD	4*x*	8	2	2			2		1
								5*x*	4	3	2	2				
								7*x*	8	5	3	3				1
69	*P. puberula*	45.74933	10.87946	Avio 1210	I	1210	FDN & AT	5*x*	12	3	1	1				3
								7*x*	1	1						1
70	*P. puberula*	46.02592	10.97567	Lasino	I	490	FDN & AT	4*x*	19	4	4			4		
								6*x*	2	2						
71	*P. puberula*	45.61144	10.36253	San Eusebio	I	630	HAM, CD & FDN	4*x*	20	4	2			2		
72	*P. puberula*	45.65391	10.43776	Pavone	I	330	HAM, CD & FDN	4*x*	12	4	3			3		

Appendix 1. Continued..

ID	Taxon	Lat. (°N)	Long. (°E)	Population	Country[a]	Elevation (m a.s.l.)	Collectors[b]	Ploidy levels	N	N_g	N_s	A	HP	S_{reg}	S_{irreg}	N_i
73	*P. puberula*	45.59223	10.87572	Forte Masua	I	960	HAM & CD	4*x*	12	4	4			4		
74	*P. puberula*	45.77776	10.907799	S. Valentino	I	1270	FDN & AT	5*x*	4	4	2	2				
								7*x*	2	2	1			1		
75	*P. puberula*	45.74151	11.06998	Ronchi	I	800	HAM, CD & FDN	7*x*	12	7	9	6		1	2	
76	*P. puberula*	45.73367	10.93042	Avio	I	180	AT	4*x*	14	4	3			3		4
77	*P. puberula*	45.7554	11.02516	AlaE	I	350	AT	4*x*	18	3	2			2		
78	*P. puberula*	46.27797	11.43325	Castellano	I	950	FDN & AT	4*x*	11	4	2			2		3
								6*x*	1	1	3			3		1
79	*P. puberula*	45.85773	10.93782	Santantonio	I	360	HAM & CD	4*x*	12	3	2			2		
80	*P. puberula*	45.80342	10.9103	S. Giacomo	I	1360	HAM & CD	5*x*	6	3	1	1				3
								6*x*	1	1	1	1				1
								7*x*	4	2	2	2				2
								8*x*	3	2	6	5	1			2
81	*P. puberula*	46.43081	11.24233	Terlan Mendel	I	600	HAM & FDN	4*x*	19	2	2			2		
82	*P. puberula*	46.35469	11.243	Söll	I	360	HAM & FDN	4*x*	19	3	1			1		
83	*P. puberula*	46.27308	11.18047	Unterfennberg	I	1050	HAM & FDN	5*x*	15	4	1	1				
								7*x*	4	4	4	4				
84	*P. puberula*	46.22039	11.29131	Grumes	I	890	FDN & AT	5*x*	6	4	2	2				2
								6*x*	9	4	2	2				1
								7*x*	3	3	1	1				2
85	*P. puberula*	46.24878	11.24806	Buchholz	I	550	FDN & AT	5*x*	7	4	1			1		2
								7*x*	7	4	3	3				2
								8*x*	6	4	7	4	3			2
86	*P. puberula*	46.65114	11.2195	Hafling Oberdorf	I	1340	HAM & FDN	5*x*	7	4	1	1				2
								7*x*	11	4	3	3				3
								8*x*	1	1	1	1				1
87	*P. puberula*	46.83247	11.16847	Moos Passeier	I	1050	HAM & FDN	5*x*	16	3	3	3				2
								7*x*	6	4	2	2				2
88	*P. puberula*	46.82269	11.19567	Passeier-Moos	I	890	HAM & FDN	5*x*	12	4	3	3				1
								6*x*	2	2	1	1				1
89	*P. puberula*	46.6992	11.16506	Finele	I	570	HAM & FDN	4*x*	14	4	4			4		
90	*P. puberula*	46.74197	10.82583	Fineil	I	1975	HAM & FDN	5*x*	4	4	1	1				2
								7*x*	4	4	2	1		1		
91	*P. puberula*	46.73864	10.85089	Vernagt	I	1760	HAM & FDN	4*x*	10	3	2			2		3
								6*x*	1	1						1
92	*P. puberula*	46.706	10.91764	Pfossertal Abzw	I	1250	HAM & FDN	4*x*	11	4	3			3		4
								6*x*	1	1	3			3		1
93	*P. puberula*	46.83958	10.65644	Melag	I	1940	HAM & FDN	4*x*	13	4	4			4		
94	*P. puberula*	46.66813	10.58537	Schluderns	I	1010	HAM & FDN	4*x*	14	4	3			3		
95	*P. puberula*	46.81395	10.57831	Perdross	I	1700	HAM & FDN	4*x*	12	4	2			2		4
								5*x*	2	2	3			3		2
96	*P. puberula*	46.43325	11.54412	Welschnofen	I	1200	HAM & FDN	5*x*	4	3	2	2				
								7*x*	16	4	3	3				
97	*P. puberula*	46.47972	11.30494	Sigmundskron	I	340	HAM & FDN	4*x*	14	4	4			4		
98	*P. puberula*	46.40467	11.45053	Schwarzenbach	I	1410	HAM & FDN	5*x*	2	2	2	2				
								7*x*	11	2	1	1				1
								8*x*	2	2	2	2				
99	*P. puberula*	46.37103	11.35436	Aldein	I	1225	HAM & FDN	5*x*	21	3	3	3				
								8*x*	1	1	1		1			
100	*P. puberula*	46.75	10.51728	Zerzertal	I	1810	HAM & FDN	4*x*	12	3	4			4		3
								5*x*	9	4	1		1			2
								7*x*	1	1						1
101	*P. puberula*	46.63458	10.76367	Kortsch	I	970	HAM & FDN	4*x*	12	4	2			2		
102	*P. puberula*	46.70395	11.12447	Hochmuth	I	1450	HAM & FDN	4*x*	12	2	2			2		
103	*P. puberula*	46.53036	11.18589	Nals	I	950	HAM & FDN	5*x*	10	4	2	1	1			
								7*x*	6	4	4	3		1		
								8*x*	3	2	2	2				
104	*P. puberula*	46.65972	10.45611	Muenstertal Tellahöfe	I	1750	HAM & FDN	4*x*	16	4	1			1		4
								6*x*	1	1						1

Appendix 1. Continued..

ID	Taxon	Lat. (°N)	Long. (°E)	Population	Country[a]	Elevation (m a.s.l.)	Collectors[b]	Ploidy levels	N	N_g	N_s	A	HP	S_{reg}	S_{irreg}	N_i
105	*P. puberula*	46.81742	10.3495	Sent	CH	1330	HAM & FDN	*4x*	19	4	3			3		
106	*P. puberula*	46.89417	10.51028	Nauders	A	1500	HAM & FDN	*4x*	20	3	3			3		
107	*P. puberula*	46.77347	10.20403	Ardez	CH	1460	HAM & FDN	*4x*	10	4	4			4		
108	*P. puberula*	46.99	10.565	Lafairs	A	1100	HAM & FDN	*4x*	11	4	4			4		2
109	*P. puberula*	46.91546	11.3093	Ridnaun	I	1390	CD	*4x*	12	3	4			4		
110	*P. puberula*	46.96975	11.33019	Pflersch Ende	I	1450	CD	*5x*	10	3	1	1				
								8x	1	1	1	1				
111	*P. puberula*	46.96069	11.54327	Kematen	I	1500	CD	*5x*	8	2	2	2				1
								6x	6	2	2	2				2
								7x	3	3	2	2				2
								8x	2	2						1
112	*P. puberula*	46.87856	11.44342	Reifenstein	I	960	CD	*4x*	12	3	4			4		3
113	*P. puberula*	46.91411	11.46227	Flans	I	1430	CD	*4x*	12	4	4			4		
114	*P. puberula*	46.58533	11.53644	Trostburg	I	1010	CD	*4x*	16	4	4			4		
								7x	4	3	2	2				
115	*P. puberula*	46.5842	11.63529	St. Ulrich	I	1130	CD	*5x*	8	3	2	2				
								6x	1	1	1	1				
								7x	11	4	5	4		1		
116	*P. puberula*	46.67389	11.55543	Latzfons	I	1280	CD	*4x*	12	3	3			2	1	
117	*P. puberula*	46.65169	11.58138	Saeben N	I	730	CD	*4x*	3	3	3			3		3
								5x	4	4	1	1				2
								7x	11	4	2	2				2
118	*P. puberula*	46.66362	11.60049	Feldthurns	I	810	CD	*4x*	11	4	4			4		
								6x	1	1	2			2		
119	*P. puberula*	46.77655	11.62985	Franzensfeste	I	730	CD	*5x*	6	4	1	1				
								7x	14	4	1	1				
120	*P. puberula*	46.70514	11.64574	Tschoetscher H	I	680	CD	*4x*	20	4	1			1		
121	*P. puberula*	46.74708	11.6535	Raas W	I	750	CD	*4x*	11	4	2			2		
								6x	1	1	1			1		
122	*P. puberula*	46.84502	11.62849	Vals	I	1410	CD	*4x*	8	3	2			2		3
								5x	9	1	1	1				1
								6x	1	1	3	3				
								8x	1							
123	*P. puberula*	46.81947	11.78566	St. Sigmund	I	1160	CD	*4x*	18	4	4			4		
								5x	2	2	3	3				
125	*P. puberula*	47.05372	10.35383	Sinsen	A	1290	HAM & FDN	*5x*	4	4	1	1				2
								8x	5	4	5	3		2		1
126	*P. puberula*	47.15181	10.41042	Flirsch	A	1190	HAM & FDN	*5x*	11	4	1	1				
127	*P. puberula*	47.13172	10.50642	Pians	A	910	HAM & FDN	*5x*	11	2	2	2				
128	*P. puberula*	47.07605	10.65247	Ladis	A	1150	HAM & FDN	*4x*	12	4	4			4		
129	*P. puberula*	47.13292	10.55844	Flathalpe	A	1100	HAM & FDN	*5x*	11	3	1	1				2
130	*P. puberula*	47.27242	10.91909	Silz N	A	690	HAM & FDN	*4x*	19	4	2			2		
131	*P. puberula*	47.07758	10.67508	Faggen2	A	920	HAM & FDN	*4x*	12	3	3			3		
132	*P. puberula*	47.05669	10.75383	Nufels S	A	1250	HAM & FDN	*5x*	11	3	2	2				1
133	*P. puberula*	47.01928	10.73756	Kaunertal Maut	A	1375	HAM & FDN	*5x*	12	4	2	2				
134	*P. puberula*	47.22028	10.86583	Ambach	A	850	HAM & FDN	*4x*	19	4	4			3	1	2
								6x	1	1	1			1		
135	*P. puberula*	47.08083	10.96583	Längenfeld	A	1180	HAM & FDN	*4x*	20	4	4			4		
136	*P. puberula*	46.95997	11.01301	Sölden	A	1400	HAM & FDN	*5x*	18	4	2	2				
137	*P. puberula*	47.04167	10.97861	Huben	A	1250	HAM & FDN	*5x*	12	3	3	3				
138	*P. puberula*	47.30922	10.99608	Affenhausen	A	870	HAM & FDN	*5x*	6	4	2	2				
								7x	12	3	1	1				
139	*P. puberula*	47.02547	10.30531	Versahl	A	1360	HAM & FDN	*5x*	2	1						
								7x	1							
140	*P. puberula*	47.31492	10.87171	Rossbach	A	1000	HAM & FDN	*5x*	12	2	1	1				
141	*P. puberula*	47.27785	11.23942	Zirl Ruine	A	720	HAM & FDN	*4x*	20	4	4			4		
142	*P. puberula*	47.30593	11.1253	Pettnau	A	690	HAM & FDN	*4x*	11	2	2			2		
143	*P. puberula*	47.03329	11.47236	Vinaders	A	1210	HAM, J.A.H, FDN & SS	*5x*	10	4	2	2				1
								8x	5	3	2	2				2

Appendix 1. Continued..

ID	Taxon	Lat. (°N)	Long. (°E)	Population	Country[a]	Elevation (m a.s.l.)	Collectors[b]	Ploidy levels	N	N_g	N_s	A	HP	S_{reg}	S_{irreg}	N_i
144	*P. puberula*	46.92309	11.69811	Boedenalm	I	1700	HAM, J.A.H, FDN & SS	4*x*	12	4	4			4		
								5*x*	6	3	2	2				
145	*P. puberula*	46.91885	11.77515	Zoesen	I	1720	JAH & SS	4*x*	9	2	4			4		
146	*P. puberula*	47.00096	11.99053	St. Jakob	I	1110	JAH & SS	4*x*	17	3	3			3		
								5*x*	2	2	2	2				
								6*x*	1	1	2			2		
147	*P. puberula*	47.03851	12.10801	Prettau	I	1540	JAH & SS	5*x*	16	3	2	2				2
148	*P. puberula*	47.02325	12.05884	St. Peter	I	1350	JAH & SS	4*x*	12	3	4			3	1	
149	*P. puberula*	46.94756	11.91475	Luttach	I	1070	JAH & SS	4*x*	16	2	1			1		
								5*x*	3	3	2	2				
150	*P. puberula*	46.74927	11.86294	Onach	I	1230	JAH & SS	5*x*	12	1						
								6*x*	1	1	1	1				1
								7*x*	1	1						1
								8*x*	1	1	1		1			1
151	*P. puberula*	46.69656	11.89933	Pikolein	I	1370	JAH & SS	4*x*	13	3	2			2		3
								5*x*	1	1						
152	*P. puberula*	46.78889	11.89358	Sonneburg	I	860	JAH & SS	4*x*	10	2	1			1		
153	*P. puberula*	46.84848	11.97746	Muehlbach	I	1540	JAH & SS	4*x*	10	4	3			3		
154	*P. puberula*	46.88095	11.62192	Fanealm	I	1700	JAH & SS	5*x*	11	1	1	1				1
155[c]	*P. puberula*	46.76267	12.1135	Thurn Ruine	I	1180	JAH & SS	4*x*	11	4	3			3		
								6*x*	1	1						
156	*P. puberula*	47.0916	11.57674	Toldern	A	1600	HAM & FDN	5*x*	15	4	3	3				
								7*x*	4	3	3	3				
								8*x*	1	1	1	1				
157	*P. puberula*	47.04639	11.53583	Plenten	A	1350	HAM & FDN	5*x*	17	4	3	3				
								7*x*	1	1	1	1				
								8*x*	2	1	1	1				
158	*P. puberula*	47.12288	11.45006	Matrei B	A	1050	HAM & FDN	5*x*	1							
								7*x*	15	3	2	1	1			
159	*P. puberula*	47.11823	11.26232	Oberbergtal	A	1350	HAM & FDN	5*x*	19	4	2	2				
								6*x*	1	1	1	1				
								8*x*	1	1	1	1				
160	*P. puberula*	47.20982	11.40512	Patsch	A	960	HAM & FDN	5*x*	7	4	1	1				
								7*x*	13	3	3	3				
161	*P. puberula*	47.29489	11.48701	Thaur	A	620	HAM & FDN	4*x*	12	4	4			4		
162	*P. puberula*	47.23673	11.40614	Vill	A	850	HAM & FDN	4*x*	12	3	3			3		
163	*P. puberula*	47.29476	11.40576	Arzler Alm	A	1000	HAM & FDN	5*x*	20	4	3	3				
164[c]	*P. puberula*	46.74275	12.21366	Toblach	I	1330	JAH & SS	4*x*	11	4	3			3		
								6*x*	1	1	1			1		
165[c]	*P. puberula*	46.74742	12.36419	Unterparggen	I	1410	JAH & SS	5*x*	6	4	1	1				
								7*x*	10	4	3	3				
								8*x*	1		1	1				
169	*P. puberula*	45.5348	11.01033	Grezzana	I	250	CD	4*x*	19	4	3			3		4
								6*x*	1	1						
170	*P. puberula*	45.78665	11.43974	Mosson	I	500	CD	4*x*	14	1	1			1		
								5*x*	4	2	2	2				
								7*x*	3	2	2	2				
171	*P. puberula*	45.74177	11.38215	Santosso	I	400	CD	4*x*	20	3	3			3		
172	*P. puberula*	45.82835	11.70737	Mignano	I	200	CD	4*x*	15	4	3			3		
								5*x*	2	2	1	1				
								7*x*	2	2	1	1				
								8*x*	1	1	1	1				
173	*P. puberula*	45.98977	12.21845	Lago di Lago	I	330	CD	5*x*	6	3	1	1				
								6*x*	3	2						
								7*x*	3	3	2	2				1
								8*x*	5	4	1	1				1
174	*P. puberula*	46.28463	12.36928	Erto	I	1030	CD	5*x*	9	4	3	3				
								7*x*	10	2	2	2				
175	*P. puberula*	46.15783	12.64385	Grizzo	I	150	CD	7*x*	9	4	2	2				2

Appendix 1. Continued..

ID	Taxon	Lat. (°N)	Long. (°E)	Population	Country[a]	Elevation (m a.s.l.)	Collectors[b]	Ploidy levels	N	N_g	N_s	A	HP	S_{reg}	S_{irreg}	N_i
176[c]	*P. puberula*	46.78551	13.14602	Gemona	I	400	CD	5x	4	1						
								6x	2	2						
								7x	13	3						
179	*P. puberula*	46.57027	13.8124	Unterfederaun	A	540	CD	7x	5	4	3	3				
								8x	3	3	5	5				
180	*P. puberula*	46.50338	13.40105	St. Catarina	I	690	CD	6x	2	2	2	2				1
								8x	4	2	2	2				1
181	*P. puberula*	46.5055	13.36923	Bagni Lusnizza	I	650	CD	6x	8	4	1	1				
182	*P. puberula*	46.39924	13.24515	Roveredo	I	410	CD	6x	10	4	1	1				1
183	*P. puberula*	46.51041	13.31276	Pontebba	I	600	CD	5x	1	1	1	1				
								6x	20	1						
184	*P. puberula*	46.45248	13.37211	Chiut	I	870	CD	5x	3	1	1	1				
								6x	10	3	4	4				1
								7x	3		1	1				
								8x	2	1	1	1				1
185	*P. puberula*	46.39156	13.13437	CampioloW	I	300	CD	4x	4	4	3			3		2
								5x	2	2	4	1			3	1
								7x	1	1	1	1				1
186[c]	*P. puberula*	46.75371	12.96362	Oberdrauburg	A	730	CD	6x	14	4	1	1				2
								8x	1	1	1	1				1
189	*P. puberula*	47.78117	13.1126944	Oberegg	A	890	FDN & AT	5x	6	5						1
								7x	8	4						1
195	*P. argentea*	46.27797	11.43325	Castellano	I	950	AT & FDN	2x	6	4						
								6x	2	1						
198	*P. argentea*	46.65114	11.2195	Hafling Oberdorf	I	1340	FDN & HAM	2x	8	5						
203	*P. argentea*	48.109146	17,009172	Edelsthal	A	270	FDN	6x	7	5						
204	*P. argentea*	47.941762	16,714377	Breitenbrunn	A	190	FDN	6x	1	1						
192	*P. aurea*	46.30302	12.2754	Forca Sesarola	I	700	CD	2x[e]	4	4						
194	*P. aurea*	46.74197	10.82583	Fineil	I	2305	FDN & HAM	2x	4	5						
196	*P. aurea*	46.88095	11.62192	Vals	I	2305	CD	2x[e]	4	4						
199	*P. aurea*	47.13292	10.55844	Flathalpe	A	2350	FDN & HAM	2x[e]	4	4						
216	*P. aurea*	47.055833	12.825	Forschungstation Glocknerstraße	A	2305	AT	2x[e]	4	4						
211_aur	*P. aurea*	46.838333	11.034167	Rotmoostal	A	2310	KH	2x	2	2						
205	*P. brauneana*	47.31363889	11.39544444	Gleirschspitze	A	2206	EH, FH & SH	2x[e]	4	3						
206	*P. brauneana*	46.51683333	10.56008333	Sulden	I	2740	EH, FH & SH	2x[e]	5	5						
214	*P. brauneana*	47.311944	11.220833	Haferlekar	A	1630	KH	2x[e]	4	4						
220	*P. brauneana*	47.055833	12.825	Forschungstation Glocknerstraße	A	2305	AT	2x[e]	5	5						
193	*P. crantzii*	46.29547	12.27181	Cima dell'Albero	I	1920	CD	4x	5	4						
207	*P. crantzii*	46.51802778	10.56825	Sulden	I	2680	EH, FH & SH	7x	5	5						
211	*P. crantzii*	46.838333	11.034167	Rotmoostal	A	2310	KH	6x	3	3						
222	*P. crantzii*	47.036944	12.356111	Sajathütte	A	2475	AT	6x	1	1						
								7x	3	3						
223	*P. crantzii*	47.055833	12.825	Forschungstation Glocknerstraße	A	2305	AT	6x	3	5						
224	*P. crantzii*	45.74888889	10.88027778	Avio 1210	I	1210	AT & FDN	5x	2	2						
								6x	1	1						
hyb_104	*P. crantzii* × *P. puberula*	46.65972	10.45611	Münstertal Tellahöfe	I	1750	FDN & HAM	5x?[f]	4	4	2	2				
hyb_122	*P. crantzii* × *P. puberula*	46.84502	11.62849	Vals	I	1410	CD	5x?[f]	1	1						
hyb_132	*P. crantzii* × *P. puberula*	47.05669	10.75383	Nufels S	A	1250	FDN & HAM	5x?[f]	1	1						
hyb_139	*P. crantzii* × *P. puberula*	47.02547	10.30531	Versahl	A	1360	FDN & HAM	7x?[f]	4	4	1	1				
hyb_147	*P. crantzii* × *P. puberula*	47.03851	12.10801	Prettau	I	1540	JAH & SS	5x?[f]	1	1	1	1				
								8x?[f]	3	3	1	1				

Appendix 1. Continued..

ID	Taxon	Lat. (°N)	Long. (°E)	Population	Country[a]	Elevation (m a.s.l.)	Collectors[b]	Ploidy levels	N	N_g	N_s	A	HP	S_{reg}	S_{irreg}	N_i
hyb_150	*P. crantzii* × *P. puberula*	46.74927	11.86294	Onach	I	1230	JAH & SS	5*x*?[f]	3	3	1	1				
hyb_151	*P. crantzii* × *P. puberula*	46.69656	11.89933	Pikolein	I	1370	JAH & SS	5*x*?[f]	2	2	1	1				
hyb_154	*P. crantzii* × *P. puberula*	46.88095	11.62192	Fanealm	I	1700	JAH & SS	5*x*?[f]	1	1	1	1				
hyb_184	*P. crantzii* × *P. puberula*	46.45248	13.37211	Chiut	I	870	CD	5*x*?[f]	2	2	1			1		
hyb_224	*P. crantzii* × *P. puberula*	45.74888889	10.88027778	Avio 1210	I	1210	AT & FDN		1	1						
hyb_26	*P. crantzii* × *P. puberula*	47.01067	12.3059	Pebellalm	A	1580	JAH & SS	6*x*?[f]	1	1	1	1				
hyb_65	*P. crantzii* × *P. puberula*	45.86729	11.18823	Zoreri	I	900	CD & HAM	6*x*?[f]	3	3	3	3				
hyb_69	*P. crantzii* × *P. puberula*	45.74933	10.87946	Avio 1210	I	1210	AT & FDN	5*x*?[f]<br>7*x*?[f]	3<br>2	3<br>2	1<br>3	1<br>2		 1		
hyb_80	*P. crantzii* × *P. puberula*	45.80342	10.9103	S. Giacomo	I	1360	CD & HAM	5*x*?[f]<br>7*x*?[f]<br>8*x*?[f]	1<br>2<br>1	1<br>2<br>1	 1 	 1 				
209	*P. frigida*	46.54577778	10.42786111	Piz Umbrail	CH	2660	EH, FH & SH	4*x*[e]	4	4						
210	*P. frigida*	46.7565	10.48316667	St. Valentin	I	2825	EH, FH & SH	4*x*[e]	5	5						
213	*P. frigida*	46.84889	11.030833	Hohe Mut	A	2590	KH	4*x*[e]	4	4						
225	*P. grandiflora*	47.0325	12.37222222	Sajatmähder	A	2324	AT	4*x*[e]	3	3						
197	*P. incana*	45.52858	10.28635	Sant'Eufemia	I	1640	FDN	4*x*	3	3						
200	*P. incana*	48.127348	16,252334	Perchtoldsdorfer Heide	A	310	FDN	4*x*	5	5						
201	*P. incana*	48.120765	16,931566	Hainburg an der Donau	A	215	FDN	4*x*	5	5						
202	*P. incana*	47.941762	16,714377	Breitenbrunn	A	190	FDN	4*x*	5	5						

N: total number of individuals collected; N_g: number of individuals successfully genotyped for the AFLP analysis; N_s: number of seeds screened by FCSS; A: number of seeds derived by apomixis; HP: number of seeds derived by haploid parthenogenesis; S_{reg}: number of seeds derived by regular sexuality; S_{irreg}: number of seeds derived by irregular sexuality (B_{III} hybrids formation); N_i: number of individuals selected for the interspecific analysis. Hybridity status of *P. crantzii* × *P. puberula* individuals was determined in this study.

a A: Austria; CH: Switzerland; I: Italy

b AT: Andreas Tribsch; CD: Christoph Dobeš; EH: Elvira Hörandl; FDN: Flavia Domizia Nardi; FH: Franz Hadacek; HAM: Henar Alonso-Marcos; JAH: Julian Ananda Haider; KH: Karl Hülber; SH: Stephan Hörandl; SS: Simon Stifter

c Population from Eastern Tyrol, Austria.

d Ploidy and reproductive mode determined by Dobeš & al. (2018).

e Ploidy levels taken from the literature (Dobeš & Vitek, 2000; Kurtto & al., 2004)

f The exact ploidies of *P. crantzii* × *P. puberula* remained uncertain.

Appendix 2. Herbarium vouchers representing studied taxa and deposited in W and GOET (an extended tabular version is available as Table S1 in Electr. Suppl. 1).

Taxon, population, country, province, collection number, barcode

Potentilla argentea L., **195**, Italy, Trentino-Alto Adige, *Nardi, F.D. 195_03* (W 2018-0002034); **195**, Italy, Trentino-Alto Adige, *Nardi, F.D. 195_04* (W 2018-0002036); **195**, Italy, Trentino-Alto Adige, *Nardi, F.D. 195_05* (W 2018-0002033); **198**, Italy, Trentino-Alto Adige, *Nardi, F.D. 198_08* (W 2018-0002032); **198**, Italy, Trentino-Alto Adige, *Nardi, F.D. 198_10* (W 2018-0002031); ***P. aurea*** L., **194**, Italy, Trentino-Alto Adige, *Nardi, F.D. 194_08* (W 2018-0002038); **199**, Italy, Trentino-Alto Adige, *Nardi, F.D. 199_04* (W 2018-0002035); ***P. brauneana*** Hoppe, **205**, Austria, Tyrol, *Hörandl, E., Hörandl, S., Hadacek, F. 10247* (GOET); **206**, Italy, Trentino-Alto Adige, *Hörandl, E., Hörandl, S., Hadacek, F. 10267* (GOET); ***P. crantzii*** (Crantz) Beck ex Fritsch, **207**, Italy, Trentino-Alto Adige, *Hörandl, E., Hörandl, S., Hadacek, F. 10266* (GOET); **208**, Switzerland, Grisons, *Hörandl, E., Hörandl, S., Hadacek, F. 10278* (GOET); ***P. crantzii*** (Crantz) Beck ex Fritsch × ***P. puberula*** Krašan, **026**, Austria, Tyrol, *Nardi, F.D. 026_31* (W 2018-0002240); **065**, Italy, Trentino-Alto Adige, *Nardi, F.D. 065_12* (W 2018-0002224); **069**, Italy, Trentino-Alto Adige, *Nardi, F.D. 069_02* (W 2018-0002214); **069**, Italy, Trentino-Alto Adige, *Nardi, F.D. 069_03* (W 2018-0002247); **098**, Italy, Trentino-Alto Adige, *Nardi, F.D. 098_01* (W 2018-0002148); **104**, Italy, Trentino-Alto Adige, *Nardi, F.D. 104_07* (W 2018-0002167); **139**, Austria, Tyrol, *Nardi, F.D. 139_01* (W 2018-0002125); **147**, Italy, Trentino-Alto Adige, *Nardi, F.D. 147_07* (W 2018-0002075); **147**, Italy, Trentino-Alto Adige, *Nardi, F.D. 147_15* (W 2018-0002116); **150**, Italy, Trentino-Alto Adige, *Nardi, F.D. 150_17* (W 2018-0002112); **151**, Italy, Trentino-Alto Adige, *Nardi, F.D. 151_13* (W 2018-0002109); **174**, Italy, Friuli-Venezia Giulia, *Nardi, F.D. 174_07* (W 2018-0002084); **184**, Italy, Friuli-Venezia Giulia, *Nardi, F.D. 184_01* (W 2018-0002076); ***P. frigida*** Vill., **209**, Switzerland, Grisons, *Hörandl, E., Hörandl, S., Hadacek, F. 10277* (GOET); **210**, Italy, Trentino-Alto Adige, *Hörandl, E., Hörandl, S., Hadacek, F. 10283* (GOET); ***P. incana*** G.Gaertn., B.Mey. & Scherb., **197**, Italy, Lombardy,

Appendix 2. Continued.

Nardi, F.D. 197_06 (W 2018-0002030); **197**, Italy, Lombardy, *Nardi, F.D. 197_10* (W 2018-0002029); ***P. puberula*** Krašan, **005**, Austria, Tyrol, *Nardi, F.D. 005_54* (W 2018-0002246); **006**, Austria, Tyrol, *Nardi, F.D. 006_54* (W 2018-0002245); **006**, Austria, Tyrol, *Nardi, F.D. 006_55* (W 2018-0002244); **007**, Austria, Tyrol, *Nardi, F.D. 007_54* (W 2018-0002243); **007**, Austria, Tyrol, *Nardi, F.D. 007_62* (W 2018-0002242); **016**, Austria, Tyrol, *Nardi, F.D. 016_52* (W 2018-0002241); **022**, Austria, Tyrol, *Nardi, F.D. 022_60* (W 2018-0002236); **026**, Austria, Tyrol, *Nardi, F.D. 026_42* (W 2018-0002222); **029**, Austria, Tyrol, *Nardi, F.D. 029_44* (W 2018-0002239); **029**, Austria, Tyrol, *Nardi, F.D. 029_47* (W 2018-0002237); **029**, Austria, Tyrol, *Nardi, F.D. 029_48* (W 2018-0002238); **031**, Austria, Tyrol, *Nardi, F.D. 031_41* (W 2018-0002235); **039**, Austria, Tyrol, *Nardi, F.D. 039_45* (W 2018-0002233); **040**, Austria, Tyrol, *Nardi, F.D. 040_46* (W 2018-0002234); **040**, Austria, Tyrol, *Nardi, F.D. 040_52* (W 2018-0002232); **043**, Austria, Tyrol, *Nardi, F.D. 043_24* (W 2018-0002231); **045**, Austria, Tyrol, *Nardi, F.D. 045_34* (W 2018-0002230); **054**, Austria, Tyrol, *Nardi, F.D. 054_19* (W 2018-0002221); **061**, Italy, Trentino-Alto Adige, *Nardi, F.D. 061_07* (W 2018-0002229); **062**, Italy, Veneto, *Nardi, F.D. 062_06* (W 2018-0002228); **063**, Italy, Veneto, *Nardi, F.D. 063_06* (W 2018-0002227); **064**, Italy, Veneto, *Nardi, F.D. 064_01* (W 2018-0002223); **064**, Italy, Veneto, *Nardi, F.D. 064_09* (W 2018-0002220); **065**, Italy, Trentino-Alto Adige, *Nardi, F.D. 065_04* (W 2018-0002225); **065**, Italy, Trentino-Alto Adige, *Nardi, F.D. 065_07* (W 2018-0002226); **066**, Italy, Trentino-Alto Adige, *Nardi, F.D. 066_01* (W 2018-0002217); **066**, Italy, Trentino-Alto Adige, *Nardi, F.D. 066_06* (W 2018-0002219); **066**, Italy, Trentino-Alto Adige, *Nardi, F.D. 066_09* (W 2018-0002218); **067**, Italy, Trentino-Alto Adige, *Nardi, F.D. 067_01* (W 2018-0002216); **067**, Italy, Trentino-Alto Adige, *Nardi, F.D. 067_09* (W 2018-0002215); **068**, Italy, Lombardy, *Nardi, F.D. 068_15* (W 2018-0002213); **070**, Italy, Trentino-Alto Adige, *Nardi, F.D. 070_09* (W 2018-0002212); **071**, Italy, Lombardy, *Nardi, F.D. 071_04* (W 2018-0002048); **072**, Italy, Lombardy, *Nardi, F.D. 072_03* (W 2018-0002210); **073**, Italy, Veneto, *Nardi, F.D. 073_06* (W 2018-0002050); **074**, Italy, Trentino-Alto Adige, *Nardi, F.D. 074_02* (W 2018-0002211); **074**, Italy, Trentino-Alto Adige, *Nardi, F.D. 074_03* (W 2018-0002208); **075**, Italy, Trentino-Alto Adige, *Nardi, F.D. 075_13* (W 2018-0002069); **076**, Italy, Trentino-Alto Adige, *Nardi, F.D. 076_08* (W 2018-0002209); **078**, Italy, Trentino-Alto Adige, *Nardi, F.D. 078_08* (W 2018-0002206); **078**, Italy, Trentino-Alto Adige, *Nardi, F.D. 078_10* (W 2018-0002207); **079**, Italy, Trentino-Alto Adige, *Nardi, F.D. 079_02* (W 2018-0002049); **080**, Italy, Trentino-Alto Adige, *Nardi, F.D. 080_02* (W 2018-0002205); **080**, Italy, Trentino-Alto Adige, *Nardi, F.D. 080_03* (W 2018-0002204); **080**, Italy, Trentino-Alto Adige, *Nardi, F.D. 080_06* (W 2018-0002070); **080**, Italy, Trentino-Alto Adige, *Nardi, F.D. 080_12* (W 2018-0002202); **082**, Italy, Trentino-Alto Adige, *Nardi, F.D. 082_17* (W 2018-0002203); **083**, Italy, Trentino-Alto Adige, *Nardi, F.D. 083_02* (W 2018-0002201); **083**, Italy, Trentino-Alto Adige, *Nardi, F.D. 083_20* (W 2018-0002200); **084**, Italy, Trentino-Alto Adige, *Nardi, F.D. 084_01* (W 2018-0002199); **084**, Italy, Trentino-Alto Adige, *Nardi, F.D. 084_12* (W 2018-0002198); **084**, Italy, Trentino-Alto Adige, *Nardi, F.D. 084_16* (W 2018-0002197); **085**, Italy, Trentino-Alto Adige, *Nardi, F.D. 085_03* (W 2018-0002196); **085**, Italy, Trentino-Alto Adige, *Nardi, F.D. 085_08* (W 2018-0002195); **085**, Italy, Trentino-Alto Adige, *Nardi, F.D. 085_12* (W 2018-0002146); **086**, Italy, Trentino-Alto Adige, *Nardi, F.D. 086_19* (W 2018-0002194); **086**, Italy, Trentino-Alto Adige, *Nardi, F.D. 086_20* (W 2018-0002193); **086**, Italy, Trentino-Alto Adige, *Nardi, F.D. 086_22* (W 2018-0002192); **087**, Italy, Trentino-Alto Adige, *Nardi, F.D. 087_01* (W 2018-0002191); **087**, Italy, Trentino-Alto Adige, *Nardi, F.D. 087_20* (W 2018-0002189); **088**, Italy, Trentino-Alto Adige, *Nardi, F.D. 088_08* (W 2018-0002190); **088**, Italy, Trentino-Alto Adige, *Nardi, F.D. 088_14* (W 2018-0002188); **089**, Italy, Trentino-Alto Adige, *Nardi, F.D. 089_01* (W 2018-0002186); **090**, Italy, Trentino-Alto Adige, *Nardi, F.D. 090_02* (W 2018-0002187); **090**, Italy, Trentino-Alto Adige, *Nardi, F.D. 090_06* (W 2018-0002185); **091**, Italy, Trentino-Alto Adige, *Nardi, F.D. 091_07* (W 2018-0002184); **092**, Italy, Trentino-Alto Adige, *Nardi, F.D. 092_05* (W 2018-0002182); **092**, Italy, Trentino-Alto Adige, *Nardi, F.D. 092_10* (W 2018-0002181); **093**, Italy, Trentino-Alto Adige, *Nardi, F.D. 093_02* (W 2018-0002183); **094**, Italy, Trentino-Alto Adige, *Nardi, F.D. 094_03* (W 2018-0002180); **095**, Italy, Trentino-Alto Adige, *Nardi, F.D. 095_10* (W 2018-0002179); **096**, Italy, Trentino-Alto Adige, *Nardi, F.D. 096_02* (W 2018-0002177); **096**, Italy, Trentino-Alto Adige, *Nardi, F.D. 096_10* (W 2018-0002147); **097**, Italy, Trentino-Alto Adige, *Nardi, F.D. 097_03* (W 2018-0002178); **098**, Italy, Trentino-Alto Adige, *Nardi, F.D. 098_02* (W 2018-0002175); **098**, Italy, Trentino-Alto Adige, *Nardi, F.D. 098_07* (W 2018-0002176); **099**, Italy, Trentino-Alto Adige, *Nardi, F.D. 099_09* (W 2018-0002173); **099**, Italy, Trentino-Alto Adige, *Nardi, F.D. 099_18* (W 2018-0002174); **100**, Italy, Trentino-Alto Adige, *Nardi, F.D. 100_01* (W 2018-0002170); **100**, Italy, Trentino-Alto Adige, *Nardi, F.D. 100_02* (W 2018-0002172); **100**, Italy, Trentino-Alto Adige, *Nardi, F.D. 100_17* (W 2018-0002171); **101**, Italy, Trentino-Alto Adige, *Nardi, F.D. 101_09* (W 2018-0002169); **102**, Italy, Trentino-Alto Adige, *Nardi, F.D. 102_06* (W 2018-0002168); **103**, Italy, Trentino-Alto Adige, *Nardi, F.D. 103_06* (W 2018-0002166); **103**, Italy, Trentino-Alto Adige, *Nardi, F.D. 103_07* (W 2018-0002165); **103**, Italy, Trentino-Alto Adige, *Nardi, F.D. 103_10* (W 2018-0002068); **104**, Italy, Trentino-Alto Adige, *Nardi, F.D. 104_11* (W 2018-0002066); **104**, Italy, Trentino-Alto Adige, *Nardi, F.D. 104_13* (W 2018-0002046); **105**, Switzerland, Grisons, *Nardi, F.D. 105_08* (W 2018-0002047); **109**, Italy, Trentino-Alto Adige, *Nardi, F.D. 109_03* (W 2018-0002164); **110**, Italy, Trentino-Alto Adige, *Nardi, F.D. 110_01* (W 2018-0002163); **111**, Italy, Trentino-Alto Adige, *Nardi, F.D. 111_01* (W 2018-0002067); **111**, Italy, Trentino-Alto Adige, *Nardi, F.D. 111_04* (W 2018-0002161); **111**, Italy, Trentino-Alto Adige, *Nardi, F.D. 111_06* (W 2018-0002162); **111**, Italy, Trentino-Alto Adige, *Nardi, F.D. 111_15* (W 2018-0002160); **112**, Italy, Trentino-Alto Adige, *Nardi, F.D. 112_06* (W 2018-0002158); **113**, Italy, Trentino-Alto Adige, *Nardi, F.D. 113_04* (W 2018-0002159); **115**, Italy, Trentino-Alto Adige, *Nardi, F.D. 115_01* (W 2018-0002157); **115**, Italy, Trentino-Alto Adige, *Nardi, F.D. 115_03* (W 2018-0002156); **115**, Italy, Trentino-Alto Adige, *Nardi, F.D. 115_04* (W 2018-0002155); **116**, Italy, Trentino-Alto Adige, *Nardi, F.D. 116_03* (W 2018-0002154); **117**, Italy, Trentino-Alto Adige, *Nardi, F.D. 117_03* (W 2018-0002151); **117**, Italy, Trentino-Alto Adige, *Nardi, F.D. 117_04* (W 2018-0002153); **117**, Italy, Trentino-Alto Adige, *Nardi, F.D. 117_07* (W 2018-0002152); **118**, Italy, Trentino-Alto Adige, *Nardi, F.D. 118_01* (W 2018-0002150); **118**, Italy, Trentino-Alto Adige, *Nardi, F.D. 118_02* (W 2018-0002149); **119**, Italy, Trentino-Alto Adige, *Nardi, F.D. 119_15* (W 2018-0002111); **120**, Italy, Trentino-Alto Adige, *Nardi, F.D. 120_14* (W 2018-0002145); **121**, Italy, Trentino-Alto Adige, *Nardi, F.D. 121_01* (W 2018-0002065); **121**, Italy, Trentino-Alto Adige, *Nardi, F.D. 121_04* (W 2018-0002144); **122**, Italy, Trentino-Alto Adige, *Nardi, F.D. 122_05* (W 2018-0002062); **122**, Italy, Trentino-Alto Adige, *Nardi, F.D. 122_08* (W 2018-0002107); **122**, Italy, Trentino-Alto Adige, *Nardi, F.D. 122_10* (W 2018-0002143); **122**, Italy, Trentino-Alto Adige, *Nardi, F.D. 122_12* (W 2018-0002142); **123**, Italy, Trentino-Alto Adige, *Nardi, F.D. 123_08* (W 2018-0002141); **123**, Italy, Trentino-Alto Adige, *Nardi, F.D. 123_10* (W 2018-0002140); **125**, Austria, Tyrol, *Nardi, F.D. 125_11* (W 2018-0002045); **126**, Austria, Tyrol, *Nardi, F.D. 126_09* (W 2018-0002139); **127**, Austria, Tyrol, *Nardi, F.D. 127_07* (W 2018-0002138); **128**, Austria, Tyrol, *Nardi, F.D. 128_06* (W 2018-0002137); **129**, Austria, Tyrol, *Nardi, F.D. 129_11* (W 2018-0002136); **130**, Austria, Tyrol, *Nardi, F.D. 130_09* (W 2018-0002134); **131**, Austria, Tyrol, *Nardi, F.D. 131_01* (W 2018-0002135); **132**, Austria, Tyrol, *Nardi, F.D. 132_02* (W 2018-0002132); **133**, Austria, Tyrol, *Nardi, F.D. 133_09* (W 2018-0002133); **134**, Austria, Tyrol, *Nardi, F.D. 134_13* (W 2018-0002130); **134**, Austria, Tyrol, *Nardi, F.D. 134_15* (W 2018-0002131); **135**, Austria, Tyrol, *Nardi, F.D. 135_19* (W 2018-0002128); **136**, Austria, Tyrol, *Nardi, F.D. 136_06* (W 2018-0002064); **137**, Austria, Tyrol, *Nardi, F.D. 137_02* (W 2018-0002129); **138**, Austria, Tyrol, *Nardi, F.D. 138_10* (W 2018-0002126); **138**, Austria, Tyrol, *Nardi, F.D. 138_11* (W 2018-0002127); **139**, Austria, Tyrol, *Nardi, F.D. 139_03* (W 2018-0002124); **140**, Austria, Tyrol, *Nardi, F.D. 140_12* (W 2018-0002122); **141**, Austria, Tyrol, *Nardi, F.D. 141_01* (W 2018-0002123); **142**, Austria, Tyrol, *Nardi, F.D. 142_06* (W 2018-0002121); **143**, Austria, Tyrol, *Nardi, F.D. 143_05* (W 2018-0002110); **143**, Austria, Tyrol, *Nardi, F.D. 143_06* (W 2018-0002061); **145**, Italy, Trentino-Alto Adige, *Nardi, F.D. 145_01* (W 2018-0002119); **146**, Italy, Trentino-Alto Adige, *Nardi, F.D. 146_03* (W 2018-0002118); **146**, Italy, Trentino-Alto Adige, *Nardi, F.D. 146_07* (W 2018-0002063); **146**, Italy, Trentino-Alto Adige, *Nardi, F.D. 146_20* (W 2018-0002120); **148**, Italy, Trentino-Alto Adige, *Nardi, F.D. 148_05* (W 2018-0002117); **149**, Italy, Trentino-Alto Adige, *Nardi, F.D. 149_13* (W 2018-0002060); **149**, Italy, Trentino-Alto Adige, *Nardi, F.D. 149_14* (W 2018-0002058); **150**, Italy, Trentino-Alto Adige, *Nardi, F.D. 150_05* (W 2018-0002115); **150**, Italy, Trentino-Alto Adige, *Nardi, F.D. 150_13* (W 2018-0002114); **150**, Italy, Trentino-Alto Adige, *Nardi, F.D. 150_18* (W 2018-0002113); **151**, Italy, Trentino-Alto Adige, *Nardi, F.D. 151_03* (W 2018-0002059); **152**, Italy, Trentino-Alto Adige, *Nardi, F.D. 152_03* (W 2018-0002044); **153**, Italy, Trentino-Alto Adige, *Nardi, F.D. 153_08* (W 2018-0002108); **154**, Italy, Trentino-Alto Adige, *Nardi, F.D. 154_07* (W 2018-0002105); **156**, Austria, Tyrol, *Nardi, F.D. 156_01* (W 2018-0002104); **156**, Austria, Tyrol, *Nardi, F.D. 156_02* (W 2018-0002103); **157**, Austria, Tyrol, *Nardi, F.D. 157_02* (W 2018-0002106); **157**, Austria, Tyrol, *Nardi, F.D. 157_12* (W 2018-0002101); **158**, Austria, Tyrol, *Nardi, F.D. 158_07* (W 2018-0002102); **159**, Austria, Tyrol, *Nardi, F.D. 159_04* (W 2018-0002100); **159**, Austria, Tyrol, *Nardi, F.D. 159_16* (W 2018-0002099); **159**, Austria, Tyrol, *Nardi, F.D. 159_18* (W 2018-0002096); **160**, Austria, Tyrol, *Nardi, F.D. 160_07* (W 2018-0002097); **160**, Austria, Tyrol,

Appendix 2. Continued.

Nardi, F.D. 160_13 (W 2018-0002094); **161**, Austria, Tyrol, *Nardi, F.D. 161_02* (W 2018-0002042); **162**, Austria, Tyrol, *Nardi, F.D. 162_07* (W 2018-0002053); **163**, Austria, Tyrol, *Nardi, F.D. 163_01* (W 2018-0002095); **164**, Italy, Trentino-Alto Adige, *Nardi, F.D. 164_01* (W 2018-0002098); **164**, Italy, Trentino-Alto Adige, *Nardi, F.D. 164_04* (W 2018-0002057); **165**, Italy, Trentino-Alto Adige, *Nardi, F.D. 165_03* (W 2018-0002093); **165**, Italy, Trentino-Alto Adige, *Nardi, F.D. 165_11* (W 2018-0002091); **165**, Italy, Trentino-Alto Adige, *Nardi, F.D. 165_18* (W 2018-0002092); **169**, Italy, Veneto, *Nardi, F.D. 169_02* (W 2018-0002056); **169**, Italy, Veneto, *Nardi, F.D. 169_15* (W 2018-0002090); **170**, Italy, Veneto, *Nardi, F.D. 170_02* (W 2018-0002089); **170**, Italy, Veneto, *Nardi, F.D. 170_05* (W 2018-0002055); **170**, Italy, Veneto, *Nardi, F.D. 170_14* (W 2018-0002054); **171**, Italy, Veneto, *Nardi, F.D. 171_01* (W 2018-0002088); **172**, Italy, Veneto, *Nardi, F.D. 172_04* (W 2018-0002043); **172**, Italy, Veneto, *Nardi, F.D. 172_13* (W 2018-0002041); **172**, Italy, Veneto, *Nardi, F.D. 172_18* (W 2018-0002087); **172**, Italy, Veneto, *Nardi, F.D. 172_20* (W 2018-0002040); **173**, Italy, Friuli-Venezia Giulia, *Nardi, F.D. 173_03* (W 2018-0002086); **173**, Italy, Friuli-Venezia Giulia, *Nardi, F.D. 173_08* (W 2018-0002039); **173**, Italy, Friuli-Venezia Giulia, *Nardi, F.D. 173_14* (W 2018-0002037); **174**, Italy, Friuli-Venezia Giulia, *Nardi, F.D. 174_01* (W 2018-0002085); **175**, Italy, Friuli-Venezia Giulia, *Nardi, F.D. 175_06* (W 2018-0002083); **179**, Austria, Carinthia, *Nardi, F.D. 179_02* (W 2018-0002082); **179**, Austria, Carinthia, *Nardi, F.D. 179_04* (W 2018-0002081); **180**, Italy, Friuli-Venezia Giulia, *Nardi, F.D. 180_02* (W 2018-0002078); **180**, Italy, Friuli-Venezia Giulia, *Nardi, F.D. 180_06* (W 2018-0002080); **181**, Italy, Friuli-Venezia Giulia, *Nardi, F.D. 181_04* (W 2018-0002079); **183**, Italy, Friuli-Venezia Giulia, *Nardi, F.D. 183_09* (W 2018-0002052); **183**, Italy, Friuli-Venezia Giulia, *Nardi, F.D. 183_22* (W 2018-0002077); **184**, Italy, Friuli-Venezia Giulia, *Nardi, F.D. 184_06* (W 2018-0002074); **185**, Italy, Friuli-Venezia Giulia, *Nardi, F.D. 185_01* (W 2018-0002073); **185**, Italy, Friuli-Venezia Giulia, *Nardi, F.D. 185_05* (W 2018-0002072); **186**, Austria, Carinthia, *Nardi, F.D. 186_03* (W 2018-0002071); **186**, Austria, Carinthia, *Nardi, F.D. 186_05* (W 2018-0002051)
